# Artificial intelligence-predicted ECG age gap as a biomarker: bias-adjusted correlation with mortality and cardiovascular risk factors

**DOI:** 10.1093/ehjdh/ztaf137

**Published:** 2025-11-28

**Authors:** Myrte Barthels, Elisa Verhofstadt, Inigo Bermejo Delgado, Henri Gruwez, Laurent Pison, Noëlla Pierlet, Pieter Vandervoort

**Affiliations:** Limburg Clinical Research Centre/Mobile Health Unit, Faculty of Medicine and Life Sciences, Hasselt University, Martelarenlaan 42, Hasselt 3500, Belgium; Department Future Health, Ziekenhuis Oost-Limburg, Synaps Park 1, Genk 3600, Belgium; Qompium NV, Kempische steenweg 303/27, Hasselt 3500, Belgium; Data Science Institute, Hasselt University, Martelarenlaan 43, Hasselt 3500, Belgium; Data Science Institute, Hasselt University, Martelarenlaan 43, Hasselt 3500, Belgium; Limburg Clinical Research Centre/Mobile Health Unit, Faculty of Medicine and Life Sciences, Hasselt University, Martelarenlaan 42, Hasselt 3500, Belgium; Department Future Health, Ziekenhuis Oost-Limburg, Synaps Park 1, Genk 3600, Belgium; Department of Cardiology, Ziekenhuis Oost-Limburg, Synaps Park 1, Genk 3600, Belgium; Department of Cardiovascular Sciences, University of Leuven, Oude Markt 13, Leuven 3000, Belgium; Limburg Clinical Research Centre/Mobile Health Unit, Faculty of Medicine and Life Sciences, Hasselt University, Martelarenlaan 42, Hasselt 3500, Belgium; Department of Cardiology, Ziekenhuis Oost-Limburg, Synaps Park 1, Genk 3600, Belgium; Limburg Clinical Research Centre/Mobile Health Unit, Faculty of Medicine and Life Sciences, Hasselt University, Martelarenlaan 42, Hasselt 3500, Belgium; Data Science Department, Ziekenhuis Oost-Limburg, Synaps Park 1, Genk 3000, Belgium; Limburg Clinical Research Centre/Mobile Health Unit, Faculty of Medicine and Life Sciences, Hasselt University, Martelarenlaan 42, Hasselt 3500, Belgium; Department Future Health, Ziekenhuis Oost-Limburg, Synaps Park 1, Genk 3600, Belgium; Department of Cardiology, Ziekenhuis Oost-Limburg, Synaps Park 1, Genk 3600, Belgium

**Keywords:** Electrocardiogram, Deep learning, Biological age, Age prediction, Bias correction, Survival analysis

## Abstract

**Aims:**

Artificial intelligence models can estimate a person’s age from ECG. The gap between the predicted ECG age and chronological age, predicted age deviation (*PAD*), has been associated with cardiovascular risk factors and mortality. However, regression bias causes *PAD* to correlate with chronological age itself, potentially distorting these associations.

**Objectives:**

To investigate the bias introduced by age on *PAD* by comparing associations between *PAD* and a bias-corrected *PAD* (*PAD_bc_*) with cardiovascular risk factors and survival outcomes.

**Methods and results:**

ECG and cardiovascular risk data from Ziekenhuis Oost-Limburg (2002–23) were linked to mortality data from the Belgian National Registry. A neural network was trained to predict age from ECGs. *PAD_bc_* corresponded to the residual of *PAD* regressed on chronological age. Associations with risk factors were tested using *χ*^2^ and ANOVA. Survival was analysed with Kaplan–Meier curves and Cox proportional hazards models. We included 1 258 993 ECGs from 234 586 patients, split 40:10:50 into training, validation, and test sets by patient. In the test set [mean age 56.4 ± 16.9 years, mean absolute error (MAE) 7.9], *PAD* correlated with age (*r* = −0.54) and showed inverse associations with most risk factors; conversely, higher *PAD_bc_* (*r* = 0.00) was associated with higher prevalence of risk factors. Kaplan–Meier revealed that *PAD_bc_* above its MAE was linked to lower survival, whereas *PAD* showed the opposite. Multivariate Cox showed each 1-year increase in both *PAD* and *PAD_bc_* was associated with a 1.4% increased mortality hazard.

**Conclusion:**

*PAD_bc_* is associated with cardiovascular risk factors and mortality, offering an age-independent biomarker of biological ageing.

## Graphical Abstract

**Graphical Abstract:**
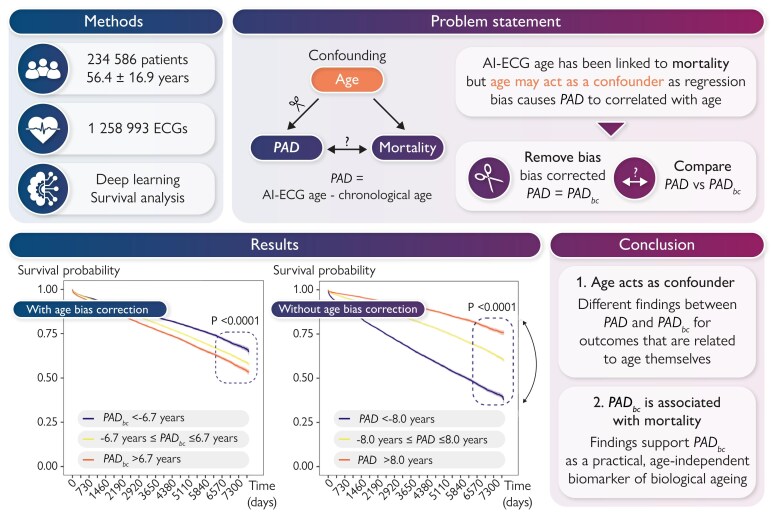

## Introduction

Deep learning for electrocardiography (ECG) signal analysis has made significant advancements in the last few years, as ECG offers a wealth of physiological information in a non-invasive and cost-effective manner.^1–8^ Among other outcomes, such as arrhythmia detection, ECG has been widely used for the detection of biomarkers related to cardiovascular diseases, as they remain the leading cause of death worldwide.^9^ One such biomarker is age, one of the most important risk factors, influencing both disease development and prognosis.^10^ Ageing has been found to affect the characteristics of the ECG, including the alteration of the QRS complex and the orthogonal *P*-wave morphology.^11–13^ Consequently, predicting the chronological age directly from a 10-s 12-lead ECG using deep learning has shown significant attention. As an example, Attia *et al*.^14^ trained a 1D convolutional neural network (CNN) on a dataset of 0.5 million ECG measurements and achieved a mean absolute error (MAE) of 6.9 years with a standard deviation (SD) of 5.6 and a correlation (*r*) of 0.84. Similar results have been obtained in datasets across different geographical locations and healthcare facilities.^5,14–19^

Interestingly, although the objective of these models was to predict the chronological age, researchers have discovered that difference between predicted ECG age and actual chronological age, predicted age deviation (*PAD*), correlates with biological ageing.^16–23^ Unlike chronological age, which simply reflects the time a person has been alive, biological age captures the cumulative effects of time, genetics, environment, lifestyle, and other factors that affect ageing. As such, it is more closely linked with the functional status of the organism.^24^ The concept of biological age offers a more encompassing overall health measure as compared to chronological age.^25^ In line with this, *PAD* seems to provide insight into the cardiovascular health of an individual, showing that ECGs reflecting a positive *PAD* of more than 6–8 years have a higher incidence of cardiovascular events compared to those with small or negative *PAD*.^14,16–18^ Furthermore, research showed an increased risk for cardiovascular and overall mortality in individuals with a positive *PAD*,^17–19,21–23^ indicating an important role of *PAD* as a predictor in overall mortality.

Although previous studies report great advancements into understanding the information embedded in *PAD*, thus far, its inherent correlation with chronological age has not been addressed. Regression models can be subject to a regression bias phenomenon, which leads to an overestimation of small values and an underestimation of large values.^26^ As the model is trained to minimize the loss function across the dataset, it tends to pull the predictions towards the central region, especially in data distributions with more data near the average. In the case of age models, younger individuals tend to get overestimated, while older individuals get underestimated, causing *PAD* to be dependent of age.^27,28^ Such correlations, both at the sample and individual levels,^29,30^ could obscure the true clinical implications of the relationship between *PAD* and clinical variables, especially when the variables of interest are also related to age.^9,31^

In this study, we aim to address this limitation by developing an ECG-based age prediction model and investigating the regression bias between *PAD* and chronological age. We correlate *PAD*, both before and after bias correction (*PAD_bc_*), with cardiovascular risk factors and survival outcomes. By correcting for the dependence on chronological age, we seek to uncover the true clinical utility of *PAD_(bc)_* and its relationship to cardiovascular risk factors, as well as its potential as a predictor of mortality. By embedding this tool within the existing care pathway, automatically analysing ECGs during standard workflows, *PAD_bc_* may offer a scalable and accessible approach to augment clinical decision-making, guide preventive interventions and improve patient management decisions without additional testing burden on patients or providers.

## Methods

### Data

#### Study design

This retrospective study used routinely collected clinical data extracted from the electronic medical record (EMR) at Ziekenhuis Oost-Limburg (ZOL) (Genk, Belgium). The dataset included 10-s 12-lead ECGs between 1 October 2002 and 31 December 2023 and was extracted from the MUSE data management system, together with ECG-derived parameters and diagnostic labels provided by the GE Marquette 12SL software. Demographic and clinical data were extracted from structured EMR records, medication history, and patient questionnaires, including cardiovascular risk and intoxication profiles. Alongside each patient’s birthdate and sex, body mass index (BMI) and four binary cardiovascular risk factors were included in this study: Smoking, diabetes, hypertension, and hypercholesterolaemia.^9^ Detailed information can be found in the supplementary materials (Supplement  *S1*). Mortality data were obtained from the Belgian National Registry. Variables such as ethnicity and socioeconomic status were not systematically available in the EMR and were therefore not included in the analyses.

This study was reviewed and approved by the medical ethics committee of Ziekenhuis Oost-Limburg. The developed model is not approved by legal authorities (e.g. CE or FDA) and was, for this study, intended solely for research purposes. The study was reported in accordance with the EHRA AI checklist to ensure transparent and reproducible reporting of AI-based prediction models in healthcare^32^ (see Supplement  *S40*).

#### Outcomes

The primary outcome of the study was the patient’s chronological age, expressed as the time between the patient’s birthdate and the ECG acquisition date. The prediction model used the raw ECG waveform data as input. The secondary outcome was all-cause mortality, modelled using survival analysis. Survival time was expressed as the time between the ECG acquisition and time of death or censoring. Right-censoring was applied if the patient was alive at the end of the observation period (maximum 7305 days or 20 years). *PAD* was evaluated as a predictor of mortality, alongside the chronological age and cardiovascular risk factors. All predictors were treated as fixed at the time of ECG acquisition, acknowledging that the risk factors may not strictly precede the ECG in time due to retrospective labelling.

#### Inclusion criteria

All patients with at least one eligible ECG during the study period were considered. ECG recordings were excluded if the patient was under 18 years of age at the time of measurement or if the software indicated a measurement failure (bad measurement quality, faulty measurement setup, inability to interpret the ECG due to lack of QRS complexes, muscle tremor, and electrode noise). Patients, along with their corresponding ECGs, were stratified for sex and age at first measurement and randomly allocated to training, internal validation, and testing datasets in a 40:10:50 ratio. Given the magnitude of the dataset size, the test set was intentionally large to support downstream statistical and survival analyses. Overlap between patients in the training, validation, and test sets was prevented by ensuring all data from the same patient appeared in only one dataset. To avoid bias caused by overrepresentation of patients with multiple ECG measurements, training and evaluation of the age prediction model only considered the first available ECG measurement within the inclusion period for each patient. The statistical and survival analysis was performed on the test set, which was further subselected by excluding patients with erroneous mortality information. Analysis concerning the risk factors only considered the first ECG taken after 2018, as ZOL switched to a cross-departmental EMR in October 2018 (most risk factors are digitally only recorded from this date onwards).

#### Dataset splitting

**Figure 1:**
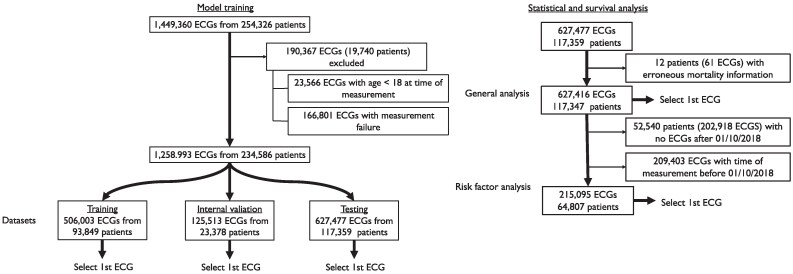
Patient flow diagram.

Data extraction from the ZOL database resulted in 1 449 360 ECG recordings from 254 326 patients. After applying the exclusion criteria, 1 258 993 ECGs from 234 586 patients remained, from which the first ECG was selected, obtaining a training set of 93 849 ECGs, a test set of 117 539 ECGs, and a validation set of 23 378 ECGs. The test set was further refined for the statistical and survival analysis by removing patients with no mortality information, resulting in 117 354 ECGs for the general analysis and 64 807 ECGs for the analysis including the risk factors (*Figure 1*).

#### Data preprocessing

The ECGs were acquired at a sampling rate of either 250 or 500 Hz (depending on the settings at the time of measurement) and were resampled to 500 Hz for further analysis. No further preprocessing was applied. Patterns of missingness within the data were examined and will be further detailed in the supplementary materials (Supplement  *S2*).

### Model

We developed a deep learning model for ECG-based age prediction, inspired by the work of Attia *et al*.^14^ The network consists of eight sequential blocks of convolutional, batch normalization, and max pooling layers to extract temporal features from the ECG signals for each lead. A spatial block aggregates information across the different leads, and two final fully connected layers together with a linear activation function produce the final age estimate. The model input consists of the raw ECG and is given by a 12 by 5000 matrix (ECG recorded for 10 s at 500 Hz) and was trained to optimize the mean squared error loss. Full architectural details and hyperparameters are provided in the supplementary materials (Supplement  *S3*).

### Evaluation

#### Age prediction model performance

In the primary analysis, the performance of the age prediction model was evaluated using the MAE, the Pearson correlation coefficient (*r*), and the intercept and the slope of a linear regression between the predicted ECG-age and the chronological age. Performance was further compared across subgroups determined by sex, age and heart rhythm (derived from the diagnostic labels provided by the 12L GE Marquette software: sinus rhythm, atrial fibrillation, and atrial flutter or other rhythms). Statistical significance was assessed using a non-parametric bootstrap test, generating 5000 different samples, and the Monte Carlo *P*-value was calculated.

Saliency mapping was used to identify electrocardiographic regions that were most influential on age prediction. The map was calculated for the median wave of all ECG leads and was averaged over a sample of 4096 patients.

#### Predicted age deviation analysis

In the secondary analysis, we examined the relationship between *PAD* and cardiovascular risk factors as well as mortality. We explicitly investigate the regression bias between the chronological age and *PAD*, as it may affect the apparent relationship between *PAD* and the risk factors of interest (BMI, smoking, arrhythmias, hypertension, hypercholesterolaemia, and diabetes) as well as mortality, as these variables are also related to chronological age.

##### Correction

A bias corrected version of *PAD* was defined as the residual of the regression of the age deviation on chronological age. The linear correlation is mitigated by imposing a correction of the data using Beheshti’s method.^29^ Given the ECG predicted age (*PA*) and the chronological age (*CA*), a fitted regression model can be expressed asPA=α+β×CA+ε . The corrected predicted age (*PA_c_)* and the corrected *PAD* (*PAD_c_*) were computed as:


$$PA_{c}\;=\;PA\;-\;(\alpha \;+\;\beta \;\times \;CA)\;+\;CA$$

$$PAD_{c}\;=\;PA_{c}\;-\;CA\;=\;PA\;-\;(\alpha \;+\;\;\beta \;\times \;CA)$$

The remaining non-linear relationship across different ages is removed using age-level bias correction (*PAD_bc_*).^30^ By subtracting the mean *PAD_c_* (*MPAD_c_*), corresponding to each integer chronological age value, from *PAD_c_* itself, any residual correlation will be eliminated.


$$MPAC_{c}(i)\;=\;\{\begin{array}{c} \begin{array}{c} \frac{1}{N_{i}}\;\overset{N_{i}}{\underset{\;j=1}{\sum }}\;PAD_{c,\;j}(i),\;for\;i\;\epsilon \;\{\min(CA),\;95\}\;\\ \frac{1}{k}\overset{k}{\underset{1}{\sum }}\frac{1}{N_{m}}\;\overset{N_{m}}{\underset{\;j=1}{\sum }}\;PAD_{c,\;j}(m),\;for\;i>95\; \end{array} \end{array}$$

$$PAD_{bc}\;=\;\;PAD_{c}\;-\;\;MPAD_{c}$$

With $N_{i}$ the total number of samples at age *i* for i≤95, *m* the unique ages for i>95, $N_{m}$ the total number of samples at age *m* and *k* the number of unique ages when i>95. *MPAD_c_* for i>95 is represented by an age-band mean ({min(m),max(m)}rather than an age-level as the dataset becomes very sparse (see Supplement  *S2*, Supplementary material online, *Figure S2*) and the age-level$MPAD_{c}(i),\;for\;i>95$ becomes unstable or undefined. We assessed the effect of the bias correction by performing the risk factor and survival analysis on both *PAD* and *PAD_bc_*. Parameters *α* and *β* as well as *MPAD_c_* were estimated on the validation set.

##### Risk factor analysis

*PAD* and *PAD_bc_* were categorized into three groups: (bias corrected) underestimated age deviation (*UAD_(bc)_*), (bias corrected) small age deviation (*SAD_(bc)_*), and (bias corrected) overestimated age deviation (*OAD_(bc)_*). Group cutoffs were set based on the MAE of the validation set of the respective age deviation, *PAD*, or *PAD_bc_*. Within each group, differences in distribution of the risk factors were examined. For binary risk factors, a *χ*^2^ test was performed. For the continuous variable, a one-way ANOVA was applied. *P*-values were adjusted using the Bonferroni method to control for multiple testing.

##### Survival analysis

###### Kaplan–Meier

Survival functions were estimated for each of the *PAD*/*PAD_bc_* groups. Confidence intervals (CIs) were calculated using the log-log transformation, and group differences were assessed with the non-parametric logrank test.

###### Cox proportional hazards model

To investigate the relationship between the continuous *PAD*/*PAD_bc_* and survival, a Cox proportional hazards model was fitted adjusting for chronological age, termed simple model. A second model, termed risk factor model, included the risk factors (BMI, smoking behaviour, hypercholesterolaemia, hypertension, heart rhythm, and diabetes) as additional covariates to evaluate the incremental effect of *PAD*/*PAD_bc_* when these factors are known.

We applied diagnostic methods to evaluate the assumptions underlying the Cox model. The functional form of the Martingale residuals with respect to the covariates of interest were examined to assess whether each covariate maintained a linear relationship with the log hazard; non-random patterns or curvature in the residuals would indicate the need for transformation or alternative modelling approaches. Schoenfeld residual plots were used to detect any systematic relationship between the covariates and time, which would suggest a violation of the proportional hazards assumption. In addition, plots of the log(-log(survival)) function against log(time) were used to visually evaluate proportional hazards; non-parallel curves would suggest that hazard ratios (HRs) vary over time. Finally, model goodness-of-fit was assessed using Cox–Snell residuals. A close alignment between the cumulative hazard of these residuals and the 45-degree reference line suggests an adequate model fit, while systematic deviations indicate potential misspecification.

## Results

### Data

Baseline patient demographics of the train, internal validation, and test set, as well as for the survival analysis with and without risk factors, are available in the supplementary materials (see Supplementary material online, *Tables S1* and *S2*). Missing data were observed for smoking and BMI and were assumed to follow a missing at random mechanism, supported by observed associations with other recorded variables. Multiple imputation using predictive mean matching was performed to account for missingness. Full details of the imputation strategy, diagnostics, and sensitivity analyses are provided in the supplementary materials (Supplement  *S2*).

### Age prediction model performance

**Figure 2:**
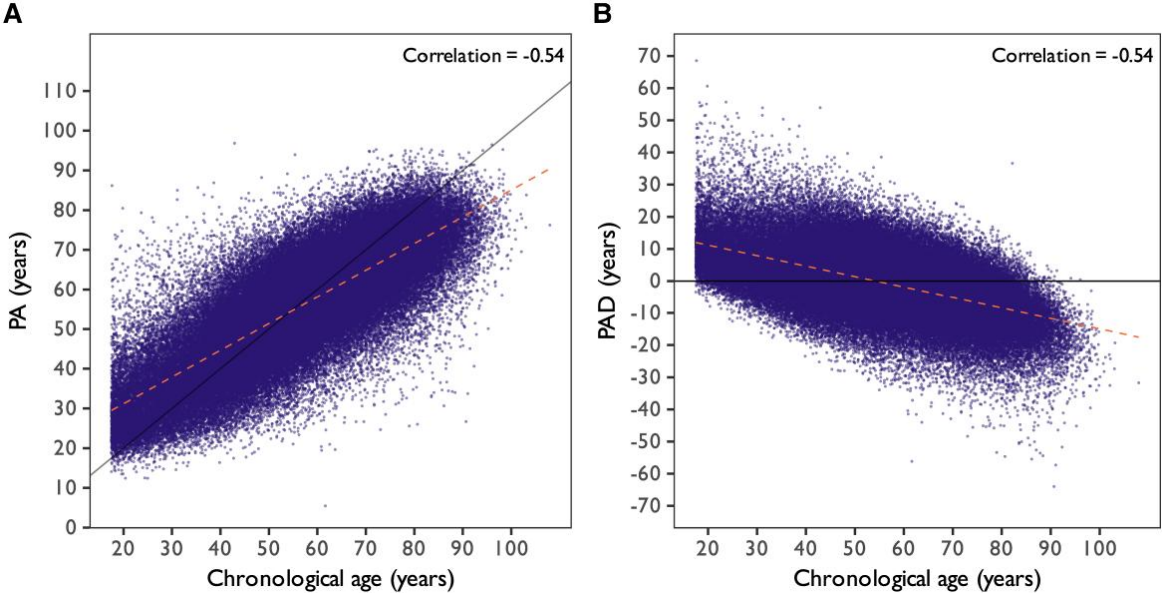
Scatter plot of (*A*) ECG predicted age and (*B*) predicted age deviation by chronological age. The diagonal and the horizontal line represent the exact mapping. The orange dotted line represents the regression line found in the data.

Model performance was evaluated using the MAE and Pearson correlation coefficient (*r*) across the training (MAE: 5.8 ± 4.5 years, *r*: 0.90), internal validation (MAE: 8.0 ± 6.4 years, *r*: 0.79), and test sets (MAE: 7.9 ± 6.4 years, *r*: 0.80). The model was moderately overtrained on the trainset. Scatter plots of the chronological age vs. *PA* and *PAD* derived from the test set are presented in *Figure 2*. A systematic deviation is observed, with a negative correlation between the chronological age and *PAD* (*r* = −0.54), consistent with the regression bias, overestimating age in younger patients and underestimating in older patients.

Subgroup performance metrics are shown in *Table 1*, including MAE stratified by sex, age group, and heart rhythm category. Statistical significance was assessed using a non-parametric bootstrap with 5000 samples. MAE was significantly lower in male patients compared to female patients. The age subanalysis confirmed the regression bias: both younger patients (<35 years) and older patients (>85 years) exhibited higher MAEs than patients closer to the mean age of the test set. Finally, patients with sinus rhythm had the lowest MAE, increasing for those with atrial fibrillation, atrial flutter, or other arrhythmias. The saliency map reveals that the *P* wave is most influential on the age prediction (see Supplementary material online, *Figure S1*).

**Table 1:** Mean absolute error (MAE) and standard deviation (SD) across different subgroups

	MAE	SD
*Gender*		
Male	7.81	6.28
Female	8.07	6.51
*Age*		
<35 years	9.28	7.81
35–60 years	7.25	5.67
60–85 years	7.79	6.19
>85 years	14.8	7.90
*Heart rhythm*		
Sinus	7.86	6.31
Atrial fibrillation	8.94	7.10
Other	9.45	7.77

### Age deviation analysis

#### Correction

**Figure 3:**
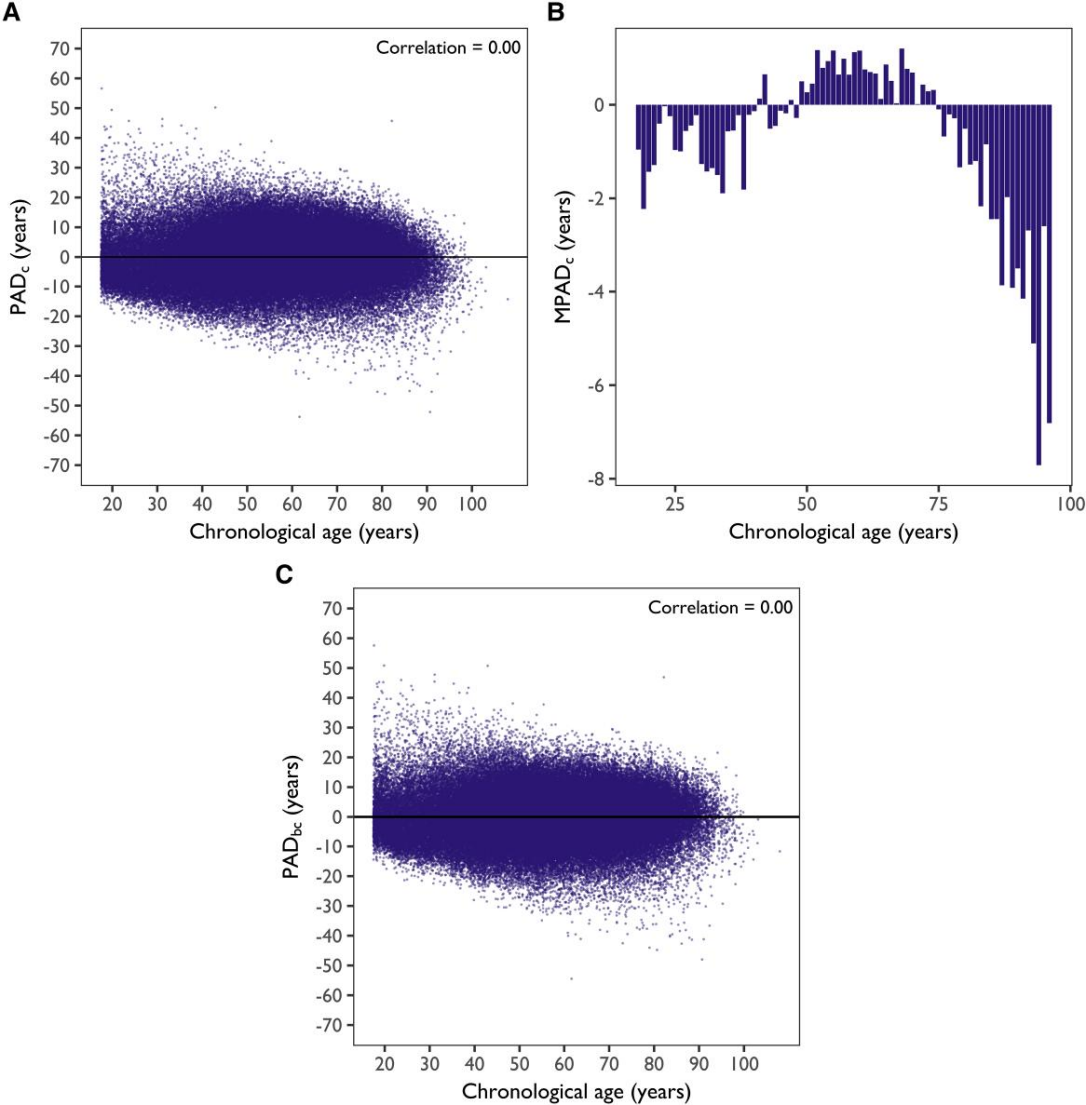
(*A*) Scatter plot of sample-level corrected age deviation (*PAD_c_*) by chronological age. (*B*) Mean age-level bias of age deviation (*MPAD_c_*) by chronological age after sample-level correction. (*C*) Scatter plot of sample-level and individual-level corrected age deviation (*PAD_bc_*) by chronological age.

Based on the regression results, *PAD_c_* was obtained using Beheshti’s method, accounting for the sample level correlation (*α* = 17.7, *β* = 0.675, *Figure 3A*). *MPAD_c_* revealed the remaining non-linear relationship across ages (*Figure 3B*) and was further subtracted from *PAD_c_* to obtain *PAD_bc_* (validation set MAE: 6.7 ± 5.3 years; test set MAE: 6.6 ± 5.3 years; *Figure 3C*).

#### Risk factors analysis

*Table 2* represents the distribution of the risk factors across the *PAD* and *PAD_bc_* groups, respectively, for the complete case per comorbidity. Significant differences (*P* < 0.001) were observed across the different groups, with mostly opposing trends between *PAD* and *PAD_bc_*. For both *PAD* and *PAD_bc_*, mean BMI and smoking prevalence increased from the underestimated age deviation (*UAD_(bc)_*) to overestimated (*OAD_(bc)_*) groups. However, for *PAD_bc_*, the prevalence of diabetes, hypertension and hypercholesterolaemia, and non-sinus rhythms increased or remained unchanged across the same groups, while a decreasing trend was seen in *PAD*. This discrepancy likely reflects confounding by chronological age, which is correlated with these variables (*r* = 0.17 for diabetes, *r* = 0.37 for hypertension, *r* = 0.29 for hypercholesterolaemia and *r* = −0.17 for sinus rhythm), but not with BMI (*r* = −0.01) or smoking (*r* = −0.03). These findings were confirmed after accounting for the missingness in BMI and smoking (see Supplement  *S2*, Supplementary material online, *Figures S4* and *S5* and Supplementary material online, *Tables S6* and *S7*).

**Table 2:** Presence of risk factors for the different bias corrected predicted age deviation (*PAD_bc_*) groups and predicted age deviation (*PAD*) groups (represented after the /)

Variables	Unit	*UAD_bc_*/*UAD*	*SAD_bc_*/*SAD*	*OAD_bc_*/*OAD*	Test statistic *PAD_bc_*/*PAD*	*P*-value *PAD_bc_*/*PAD*
Patients	% *n*	21.0/21.7	58.8/59.5	20.1/18.7	—	—
		13 634/14 085	38 128/38 574	13 045/12 148	—	—
BMI	mean + std	26.5 ± 4.93/	27.5 ± 5.15/	28.2 ± 5.76/	*F*: 314.3/332.7	<0.001/<0.001
		26.5 ± 4.73	27.5 ± 5.17	28.2 ± 5.95		
Diabetes	%	19.4/24.0	22.0/22.3	26.8/20.9	$\chi^{2}$ : 219.8/36.3	<0.001/<0.001
Hypertension	%	55.2/69.6	63.3/62.4	69.7/56.6	$\chi^{2}$ : 601.3/475.6	<0.001/<0.001
Hypercholesterolaemia	%	43.1/52.2	47.3/48.2	49.6/38.1	$\chi^{2}$ : 129.1/556.1	<0.001/<0.001
Smoking	%	43.4/40.8	46.2/47.2	48.5/48.7	$\chi^{2}$ : 66.9/202.2	<0.001/<0.001
Sinus rhythm	%	97.4/94.1	95.8/95.6	90.1/94.1	$\chi^{2}$ : 874.4/75.3	<0.001/<0.001

*P*-values are corrected using the Bonferroni method. *n*, total number of patients. *UAD_bc_*, *SAD_bc_*, and *OAD_bc_*, (bias corrected) underestimated, small, and overestimated age deviation, respectively.

#### Survival analysis

##### Kaplan–Meier

**Figure 4:**
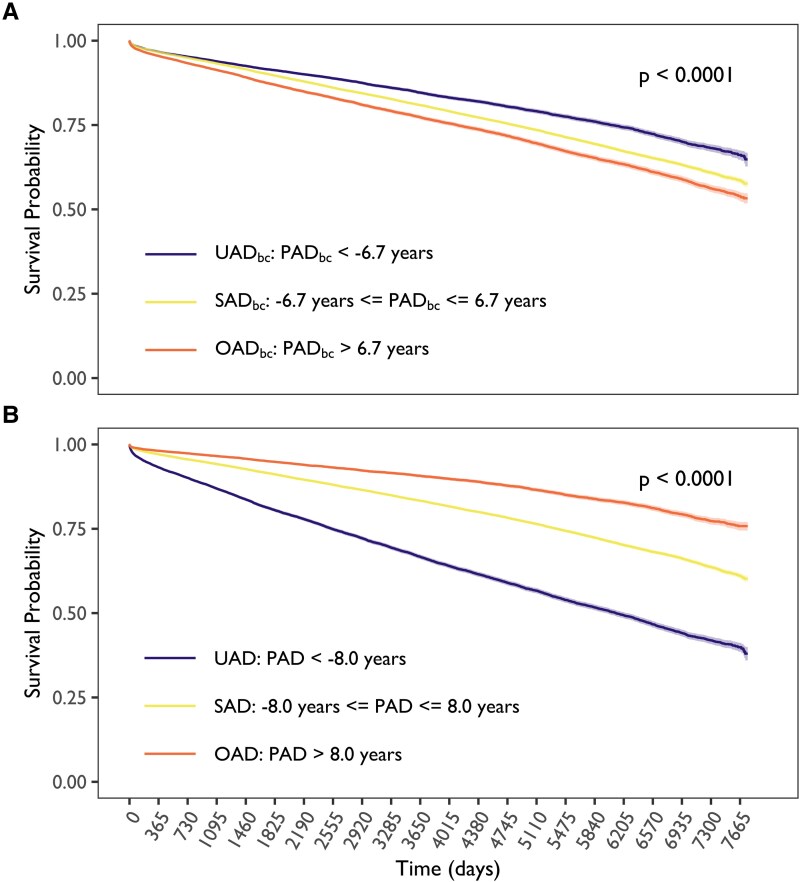
Kaplan–Meier estimates for the (*A*) corrected and (*B*) uncorrected predicted age deviation (*PAD_bc_* and *PAD*) groups: underestimated (purple, *UAD_bc_* and *UAD*), small (yellow, *SAD_bc_* and *SAD*), and overestimated age deviation (orange, *OAD_bc_* and *OAD*). The *P*-value refers to the log-rank test for difference in survival between groups.

Diverging patterns between *PAD* and *PAD_bc_* groups were observed when modelling survival using Kaplan–Meier curves. *PAD_bc_* (*Figure 4A*) showed significantly better survival in the *UAD_bc_* group compared to the *OAD_bc_* group (*P* < 0.001). In contrast, *PAD* (*Figure 4B*) showed the reverse trend, highlighting confounding by chronological age (*r* = 0.45 with mortality).

##### Cox proportional hazards model

Contrary, Cox proportional hazards models showed a general agreement between both *PAD* and *PAD_bc_* results. In the simple model, adjusting only for chronological age, both *PAD* and *PAD_bc_* were significantly associated with increased mortality risk (*Table 3*). *PAD_bc_* demonstrated a HR of 1.020 per year (95% CI: 1.018–1.021, *P* < 0.001), corresponding to a 2.0% increase in risk per unit increase, while *PAD* showed a similar HR of 1.019 (95% CI: 1.017–1.021, *P* < 0.001). The effect of chronological age was modelled using a piecewise linear transformation (age ≤ 50 and > 50 years), based on diagnostics of the functional form using martingale residuals and model comparison using AIC (see Supplementary material online, *Table S3*). The *PAD_bc_* model showed that for patients under 50, each year of age increased risk by 7.7% (HR = 1.077), compared to 10.2% for those over 50 (HR = 1.102). Similar results were found for *PAD*. Lastly, model discrimination was good, with a *C*-index of 0.812 (SE: 0.001) for both *PAD* and *PAD_bc_* and proportional hazards assumptions were met with no substantial violations (see Supplement  *S5*, Supplementary material online, *Figures S9*–S18).

**Table 3:** Cox proportional model based on chronological age and the corrected predicted age deviation (*PAD_bc_*) (above) as well as the predicted age deviation (*PAD*) (below)

Variables	HR	95% CI	*P*-value
*PAD_bc_*	1.020	1.018, 1.021	<0.001
Chronological age (≤50)	1.077	1.073, 1.081	<0.001
Chronological age (>50)	1.102	1.100, 1.103	<0.001
*PAD*	1.020	1.018, 1.021	<0.001
Chronological age (≤50)	1.082	1.078, 1.086	<0.001
Chronological age (>50)	1.111	1.110, 1.113	<0.001

95% CI, 95% confidence intervals; HR, hazard ratio.

In the risk factor Cox models, adjusting for additional cardiovascular risk factors using multiple imputations (*Table 4*), the association with *PAD_bc_* remained significant (HR: 1.014), with a *C*-index of 0.838, indicating that *PAD_bc_* captures mortality risk beyond established cardiovascular risk factors. Chronological age retained a strong association with mortality, increasing by 5.1% and 12.0% per year for those below and above 50, respectively. Male sex (HR: 1.267), smoking (HR: 1.490), diabetes (HR: 1.688), hypertension (HR: 1.450), and non-sinus rhythm (HR: 1.259) were also significantly associated with higher mortality. Interestingly, higher BMI was associated with lower mortality risk (HR: 0.956), and hypercholesterolaemia was inversely related to risk (HR: 0.574). Results were nearly identical for *PAD*, confirming that once chronological age is accounted for, residual age-related bias is minimized. Complete case model diagnostics and imputation analyses are presented in the supplementary materials (Supplement  *S5*, Supplementary material online, *Figures S19*–S21, and Supplementary material online, *Tables S8*–S13).

**Table 4:** Cox proportional hazards model based on chronological age, patient risk factors, and the bias corrected predicted age deviation (*PAD_bc_*) (above) as well as the predicted age deviation (*PAD*) (below)

Variables	HR	95% CI	*P*-value
*PAD_bc_*	1.014	1.010, 1.017	<0.001
Sex (M)	1.267	1.197, 1.341	<0.001
BMI	0.956	0.950, 0.962	<0.001
Smoking	1.490	1.406, 1.578	<0.001
Diabetes	1.688	1.597, 1.784	<0.001
Hypertension	1.450	1.326, 1.586	<0.001
Hypercholesterolaemia	0.574	0.543, 0.608	<0.001
Heart rhythm (non-sinus)	1.259	1.157, 1.369	<0.001
Chronological age (≤50)	1.051	1.043, 1.059	<0.001
Chronological age (>50)	1.120	1.116, 1.124	<0.001
*PAD*	1.013	1.009 1.016	<0.001
Sex (M)	1.266	1.196, 1.339	<0.001
BMI	0.955	0.949, 0.962	<0.001
Smoking	1.490	1.406, 1.580	<0.001
Diabetes	1.690	1.599, 1.787	<0.001
Hypertension	1.453	1.328, 1.589	<0.001
Hypercholesterolaemia	0.559	0.543, 0.608	<0.001
Heart rhythm (non-sinus)	1.266	1.164, 1.377	<0.001
Chronological age (≤50)	1.054	1.046, 1.062	<0.001
Chronological age (>50)	1.126	1.122, 1.130	<0.001

95% CI, 95% confidence intervals; HR, hazard ratio.

## Discussion

In this study, we investigated whether the difference between predicted ECG age and true chronological age, termed *PAD*, could serve as a proxy for biological ageing and cardiovascular health, while explicitly addressing the confounding effect introduced by its correlation with chronological age. Although previous research has already demonstrated that *PAD* is associated with increased cardiovascular risk and mortality, the impact of the regression bias, causing the correlation between the age deviation and chronological age, has largely been overlooked and could obscure true clinical value.

We developed a deep learning-based model to predict chronological age from 12-lead ECGs, achieving an age prediction performance (MAE: 7.9 ± 6.4 years) comparable to state-of-the-art ECG-based models.^14–16,19^ Performance varied slightly between sexes, while higher errors were observed in non-sinus rhythm groups. Saliency map analysis suggested a possible explanatory mechanism: the *P* wave was indicated as most influential on the age prediction. Its absence, a hallmark of atrial fibrillation,^33^ likely contributed to the reduced performance. Similar results were shown in Ott *et al*.^34^ Larger prediction errors occurred in the youngest (<30 years) and oldest (>85 years) participants, alongside a pronounced negative correlation between chronological age and *PAD* (*r* = −0.54), indicating the regression bias in the model.^26^ Although this bias does not invalidate the neural network’s predictions, it may confound downstream analyses, particularly when relating *PAD* to outcomes that are themselves age-dependent. To better understand and quantify this effect in the context of cardiovascular disease and mortality, we applied a bias correction to remove both linear and non-linear associations between *PAD* and chronological age.

We then compared the distribution of cardiovascular risk factors across subgroups defined by *PAD* and *PAD_bc_*. Higher *PAD_bc_* was associated with increased prevalence of hypertension, hypercholesterolaemia, diabetes, and non-sinus rhythm diagnosis, whereas opposite trends were observed for *PAD*. Notably, each of these risk factors was itself found to be correlated with age. Both *PAD* and *PAD_bc_* were associated with higher BMI and increased prevalence of smoking, factors that are largely independent of age. These results suggest that *PAD_bc_* provides a more biologically meaningful value of declining cardiovascular health than *PAD*. They further illustrate the potential of misleading or contradictory associations when *PAD* is interpreted without adjusting for its age dependency.

Survival analysis using Kaplan–Meier estimates further highlighted the confounding impact of age. Both *PAD* and *PAD_bc_* showed associations with mortality; however, the direction of association was reversed. Higher *PAD* was paradoxically associated with improved survival, while increasing *PAD_bc_* was associated with reduced survival, demonstrating a more consistent and interpretable relationship with survival, independent of age. Interestingly, in Cox proportional hazards models, where chronological age could be included as a covariate, *PAD* and *PAD_bc_* yielded similar results.

Previous research has demonstrated associations between *PAD* and CV risk factors and mortality. Similar to this work, Hirota *et al*. applied a linear bias correction and found that a *PAD_c_* > 6 years had a reduced survival probability of 7.35% at 3 years compared to a negative *PAD_c_* < −6 years of 5.23%.^35^ In other studies, lower survival probability was found for *PAD* > 6–9 years with survival curves directly adjusted for age and sex.^17,18^  ^,23,19,21^ In contrast, our analysis reports *PAD* without age adjustment, to better isolate and interpret the intrinsic value of *PAD* itself. To enable comparison with prior research, supplementary materials include age-adjusted survival curves across the *PAD* groups (see Supplement  *S4*, Supplementary material online, *Figures S6*–S8). Consistent with earlier findings, our age-adjusted survival curves show better survival in the *UAD* group compared to the *OAD*.

Our findings align with previous work in the brain age literature, where similar bias correction strategies have been recommended^28,31,35^: (1) regressing out chronological age from age deviation or (2) including age as a covariate in downstream analyses. We advocate the same for ECG-based age metrics to ensure their valid interpretation for future clinical analysis.

These results highlight that, when adequately modelled, *PAD_bc_* is a biologically informative measure, attempting to capture the physiological divergence between an individual's apparent biological age and their chronological age. Unlike traditional biological age estimations that require the integration of multiple categorical or continuous variables,^36^  *PAD_bc_* offers a single, interpretable marker directly extracted from a widely available, non-invasive, low-cost clinical tool. The ECG, currently used for acute diagnosis, may thus also serve as a scalable biomarker of biological ageing.

### Limitations

This study is best understood in terms of its limitations. First, the dataset used for model development and analysis was collected from a single tertiary care centre. As such, findings may not generalize to broader or more diverse populations. Additionally, as variables such as ethnicity and socioeconomic status were not systematically available, fairness analysis was precluded, which could potentially conceal performance disparities across underrepresented populations. Furthermore, medication effects were not accounted for in this analysis, as the data acquisition in this retrospective study did not allow for granularity between disease diagnosis and treatment exposure (see supplement  *S4*). Certain cardiovascular medications could influence ECG morphology and, by extension, model predictions for medication-specific populations. Structure data collection and external validation across diverse populations and healthcare settings would allow to identify underperforming subpopulations. By increasing the representation of these subgroups in the training group, the generalizability of the model would increase. Techniques such as Federated Learning could be adopted to access this sensitive data in a privacy-preserving way.^37^ Second, while bias correction was applied to account for its dependency on chronological age, the correction itself is influenced by the accuracy of the prediction model. In settings where model performance is suboptimal, the correlation between age and *PAD* may be artificially inflated, potentially leading to overcorrection or misinterpretation.^38^ Third, while consistent risk factor associations lend credibility to the findings, the lack of ground truth for the age deviations makes validation challenging.^32,35,39^ Lastly, the timing and frequency of risk factor measurements relative to the ECG recording were not standardized. This temporal misalignment may have weakened the precision of associations between *PAD_(bc)_* and specific clinical characteristics. Future studies should aim to incorporate temporally aligned data by using longitudinal cohort designs with synchronized data collection, strengthening causal interpretations.

### Future directions

Future research should explore ways to enhance its clinical utility and generalizability. At the population level, it should prioritize validating *PAD_bc_* in larger and more diverse populations, ideally across multiple clinical settings and healthcare systems. At the individual level, further investigation into intra-individual variability and the temporal dynamics with respect to outcome may allow to monitor lifestyle and therapeutic intervention and define its role in preventative care and clinical decision making. Ultimately, randomized controlled trials in multiple settings will be required to determine whether using *PAD_bc_* in clinical practice improves patient outcomes or alters clinician behaviour meaningfully.^40^

## Conclusion

Our study supports previous findings associating ECG-based age prediction with both cardiovascular risk factors and mortality risk, but demonstrates that the correlation between *PAD* and chronological age can obscure its interpretation. After applying bias correction, meaningful associations were identified, especially when chronological age could not be explicitly accounted for. Future studies should focus on incorporating and validating *PAD_bc_* in more diverse populations, integrating longitudinal data, and exploring its potential role as a biomarker for biological age in personalized risk stratification and preventive care.

## Supplementary Material

ztaf137_Supplementary_Data

## Data Availability

The data underlying this article will be shared on reasonable request to the corresponding author.
