# Supplementary material for: Artificial intelligence-predicted ECG age gap as a biomarker: bias-adjusted correlation with mortality and cardiovascular risk factors
Source: Eur Heart J Digit Health. 2025 Nov 28;7(2):ztaf137. doi: 10.1093/ehjdh/ztaf137 (PMC12853119; doi:10.1093/ehjdh/ztaf137)
Supplement: ztaf137_Supplementary_Data [file ztaf137_supplementary_data.pdf]

Supplementary information

Table of Contents

Abbreviations List..7

Supplement 1: Comorbidity Selection and Definition..8

Supplement 2: Missingness Information..9

Supplement 3: Model Architecture..12

Supplement 4: Age adjusted Kaplan-Meier...13

Supplement 5: Cox Proportional Hazards Model Diagnostics and Assumption Checks...13

Supplement 6: Supplementary Figure 1: Saliency map depicting the model output gradient with respect to the ECG waveform. Saliency will be large when a small change at the input voltage leads to a large change in age prediction. Dark blue shades indicate regions with the greatest influence on the age prediction. Saliency was averaged over a random sample of 4096 individuals of the main analysis test set. The red waveform depicts the median waveform in each lead among the 4096 individuals....15

Supplement 7: Supplementary Figure 2: Age histogram for the validation and testset....16

Supplement 8: Supplementary Figure 3: Missing data pattern for all risk factors. (x%) indicates the amount of missing data for this variable.......................................17

Supplement 9: Supplementary Figure 4: Trace plots for the imputed dataset based on (A) $PADbc$ and (B) PAD. The trace plots illustrate the convergence diagnostics

for the imputed datasets. Each line represents an imputed dataset. $PAD(bc)$, (bias corrected) predicted age deviation. 18

Supplement 10: Supplementary Figure 5: density distribution of BMI values after imputation based on (A) $PADbc$ and (B) PAD. The original values are shown in blue and the imputed values shown in red. $PAD(bc)$, (bias corrected) predicted age deviation. 19

Supplement 11: Supplementary Figure 6: Age histogram for (A) different bias corrected predicted age deviation ($PADbc$) groups: $UADbc$, $SADbc$ and $OADbc$ bias corrected underestimated, small and overestimated age deviation, respectively. (B) different predicted age deviation (PAD) groups: UAD, SAD and OAD underestimated, small and overestimated age deviation, respectively. 20

Supplement 12: Supplementary Figure 7: Kaplan-Meier estimates per age band for the uncorrected predicted age deviation (PAD) groups: Underestimated (purple UAD), Small (yellow SAD), and Overestimated age deviation (orange OAD). 21

Supplement 13: Supplementary Figure 8: (A) Age histogram and (B) Kaplan-Meier estimates for the age-standardized uncorrected predicted age deviation (PAD) groups: Underestimated (purple UAD), Small (yellow SAD), and Overestimated age deviation (orange OAD). 22

Supplement 14: Supplementary Figure 9: Martingale residuals under the null model for (A) chronological age and (B) under the univariate chronological age-based (piecewise linear) model for the corrected predicted age deviation ($PADbc$)...........23

Supplement 15: Supplementary Figure 10: (A) Functional form for the bias corrected predicted age deviation ($PADbc$) modelled using restricted cubic splines (B) $PADbc$ histogram. 23

Supplement 16: Supplementary Figure 11: Martingale residuals for (A) chronological age and (B) bias corrected predicted age deviation ($PADbc$) after model fit. 24

Supplement 17: Supplementary Figure 12: Schoenfeld residual plots for (A) bias corrected predicted age deviation ($PADbc$) and (B) chronological age after model fit. 24

Supplement 18: Supplementary Figure 13: Log-cumulative hazards functions for (A) bias corrected predicted age deviation ($PADbc$) and (B) chronological age after model fit. 25

Supplement 19: Supplementary Figure 14: Density distribution of the Cox-Snell residuals for censored observations (histogram) and the Nelson-Aalen estimated cumulative hazard function against the Cox-Snell residuals for the Cox proportional model based on chronological age and the corrected predicted age deviation ($PADbc$). 26

Supplement 20: Supplementary Figure 15: Martingale residuals for (A) predicted age deviation (PAD) and (B) chronological age after model fit. 26

Supplement 21: Supplementary Figure 16: Schoenfeld residual plots for (A) predicted age deviation (PAD) and (B) chronological age after model fit. 27

Supplement 22: Supplementary Figure 17: Log-cumulative hazards functions for (A) predicted age deviation (PAD) and (B) chronological age after model fit. 27

Supplement 23: Supplementary Figure 18: Density distribution of the Cox-Snell residuals for censored observations (histogram) and the Nelson-Aalen estimated cumulative hazard function against the Cox-Snell residuals for the Cox proportional model based on chronological age and predicted age deviation (PAD). 28

Supplement 24: Supplementary Figure 19: Schoenfeld residual plots for covariates in the risk-adjusted survival model based on the bias corrected predicted age deviation ($PADbc$). 29

Supplement 25: Supplementary Figure 20: Log-cumulative hazards functions for covariates in the risk-adjusted survival model based on the bias corrected predicted age deviation ($PADbc$). 32

Supplement 26: Supplementary Figure 21: Density distribution of the Cox-Snell residuals for censored observations (histogram) and the Nelson-Aalen estimated cumulative hazard function against the Cox-Snell residuals for the risk-adjusted survival model based on the bias corrected predicted age deviation ($PADbc$). The red line represents the expected 45-degree line...34

Supplement 27: Supplementary Table 1: Patient characteristics of the model training population (*Age at first ECG). 35

Supplement 28: Supplementary Table 2: Patient characteristics of the statistical and survival analysis population (*Age at first ECG). 35

Supplement 29: Supplementary Table 3: Distribution of cardiovascular risk profile registration vs medication registration for diabetes, hypertension and hypercholesterolemia. 36

Supplement 30: Supplementary Table 4: Transformations of the chronological age variable and associated AIC of the univariate Cox proportional hazards model. 36

Supplement 31: Supplementary Table 5: Percentage of missing data in risk variables (only BMI missing, only smoking missing, and both BMI and smoking missing) in the overall sample and stratified by predicted age deviation (PAD) and bias corrected predicted age deviation ($PADbc$) groups. n total number of patients, $UAD(bc)$, $SAD(bc)$ and $OAD(bc)$ (bias corrected) underestimated, small and overestimated age deviation, respectively. 37

Supplement 32: Supplementary Table 6: Summary table showing the average BMI and smoking for different bias corrected predicted age deviation ($PADbc$) groups across multiple imputations, along with associated chi-squared and F statistics. $UADbc$, $SADbc$ and $OADbc$ bias corrected underestimated, small and overestimated age deviation, respectively. 37

Supplement 33: Supplementary Table 7: Summary table showing the average BMI and smoking for different predicted age deviation (PAD) groups across multiple imputations, along with associated chi-squared and F statistics. UAD, SAD and OAD underestimated, small and overestimated age deviation, respectively.............39

Supplement 34: Supplementary Table 8: Complete case Cox proportional hazards model based on chronological age, bias corrected predicted age deviation $PADbc$ and patient risk factors. HR, Hazard ratio and 95% CI, 95% confidence intervals. ...41

Supplement 35: Supplementary Table 9: Complete case Cox proportional hazards model based on chronological age, predicted age deviation (PAD) and patient risk factors. HR, Hazard ratio and 95% CI, 95% confidence intervals...............................41

Supplement 36: Supplementary Table 10: Complete case Cox proportional hazards model based on chronological age, bias corrected predicted age deviation ($PADbc$) and patient risk factors with interaction terms for age, sex and rhythm. HR, Hazard ratio and 95% CI, 95% confidence intervals 42

Supplement 37: Supplementary Table 11: Hazard ratios (HR) for bias corrected predicted age deviation ($PADbc$) and hypercholesterolemia comparing different approaches to label hypercholesterolemia within the risk factor model. 95% CI, 95% confidence intervals 42

Supplement 38: Supplementary Table 12: Summary table showing the average Cox proportional models across multiple imputations based on chronological age, bias

corrected predicted age deviation $PADbc$ and patient risk factors. HR, Hazard ratio and 95% CI, 95% confidence intervals. 43

Supplement 39: Supplementary Table 13: Summary table showing the average Cox proportional models across multiple imputations based on chronological age, predicted age deviation (PAD) and patient risk factors. HR, Hazard ratio and 95% CI, 95% confidence intervals. 50

Supplement 40: EHRA AI Checklist 58

References 62

Abbreviations List

ATC: Anatomical Therapeutic Chemical

CI: Confidence Interval

ECG: Electrocardiogram

EMR: Electronic Medical Record

HR: Hazard Ratio

MAR: Missing At Random

MCAR: Missing Completely At Random

MNAR: Missing Not At Random

OAD: Overestimated Age Deviation

OAD_{bc} : Bias Corrected Overestimated Age Deviation

PAD : Predicted Age Deviation

PAD_{bc} : Bias Corrected Predicted Age Deviation

PMM: Predictive Mean Matching

SAD: Small Age Deviation

SAD_{bc} : Bias Corrected Small Age Deviation

UAD: Underestimated Age Deviation

UAD_{bc} : Bias Corrected Underestimated Age Deviation

WHO: World Health Organization

ZOL: Ziekenhuis Oost Limburg

Supplement 1: Comorbidity Selection and Definition

Risk factors were gathered through combining multiple data sources from the electronic medical record (EMR) at Ziekenhuis Oost-Limburg (ZOL) (Genk, Belgium).

- BMI was calculated from height and weight measurements. If multiple values were available, the value closest in time to the Electrocardiography (ECG) recording was selected.
- Diabetes, hypertension, hypercholesterolemia: Determined based on binary registrations from the cardiovascular risk profile and/or the prescription of disease-specific medications. Drugs were identified using Anatomical Therapeutic Chemical (ATC) codes, an international classification system maintained by the World Health Organization (WHO). The ATC system categorizes medications into 5 levels (1):

  1. Main anatomical or pharmacological groups
  2. Pharmacological or therapeutic use

  3&4. Chemical, pharmacological or therapeutic subgroups

  5. Chemical substance.

  In this study, comorbidity-related medications were identified by screening for ATC codes corresponding to the level 2 class, as indicated by the prefix of the code:

  - Diabetes: ATC codes starting with A10
  - Hypertension: ATC codes starting with C02, C03, C07, C08, or C09
  - Hypercholesterolemia: ATC codes starting with C10

  The presence of a comorbidity was inferred from either the registration of the comorbidity (binary) in the risk profile or from the use of at least one medication within these ATC code ranges. This study was performed on a retrospective dataset from routine care with no protocol for data collection. This resulted in an aggregated, less granular labelling procedure as compared to a structured data collection. It was opted to combine diagnosis registration and medication use to classify presence of disease as our diagnosis registration is incomplete (the diagnosis triggering the use of medication is often registered outside the hospital, which is not accessible to the authors) (Supplementary Table 3). The authors are aware that this mixes diagnosis with treatment exposure and could induce selection and treatment effects. In the specific case of hypercholesterolemia, our models seem to indicate lower mortality rate with presence of the disease, a phenomenon which has been labelled the ‘cholesterol

paradox' (2). This might reflect a treatment bias in the data. To report on this matter as transparently as possible we have attempted to split treatment effect from diagnosis for hypercholesterolemia. Different approaches to label hypercholesterolemia were explored and compared (Supplementary Table 11). Contra intuitively, the use of medication and more specifically the use of statins led to poorer results in mortality outcome (3). Furthermore, if hypercholesterolemia was not registered in the CV risk profile but the patient took hypercholesterolemia related medication, the HR indicated a higher mortality rate. This likely reflects a confounding effect, however, as we do not have the data available to investigate this matter in more detail, we cannot draw any firm conclusions on the result.

- Smoking: Determined based on binary registrations from the cardiovascular risk profile and/or categorical registration in the intoxication profile (never smoked, passive smoking, quit smoking and active smoking). Categories are binarized to 'smoker' if a patient has been registered as 'quit' or 'smoker', else 'non-smoker'

Patients were labelled as having a risk factor if any source indicated its presence at any time. All ECGs from that patient inherited the same risk factor labels. Although this approach may misrepresent temporal relationships, it was adopted due to the scale of the data and limited resources for manual annotation.

Supplement 2: Missingness Information

The dataset includes missing data for both smoking behaviour and BMI, affecting in total 6,392 patient risk profiles. Overall, smoking behaviour is absent for 3.85% of the patients, while BMI data is missing for 7.31% of the patients. Supplementary Figure 3 provides an overview of the missing data across the different observations.

Understanding the pattern of missing data is difficult in retrospect due to the wide variety of medical practitioners, clinical departments and patient demographics involved in the ECG data acquisition process. Additionally, missing data can also result from random errors in the electronic patient platform. In the latter cases, the missing data mechanism would be classified as Missing Completely At Random (MCAR), meaning that the missing data is independent of both the observed and unobserved (missing) data. Alternatively, data may be Missing At Random (MAR), meaning that missing data is dependent of the observed data, or Missing Not At Random (MNAR), where the

missing data is dependent on both the observed and unobserved data. Under MCAR and MAR assumptions, multiple imputation can be used to generate realistic values for the missing observations, generating several independent completed datasets. The results of these datasets are combined to take into account the uncertainly of the imputation process. If the missing data mechanism is identified as MNAR, the missing data process needs to be explicitly modelled, creating a model that jointly describes the data and missingness mechanism

Multiple imputation consists of several phases. In the imputation phase multiple datasets are generated where the imputed values are based on appropriate model predictions using the available data. One of the predictive models that can be used during the imputation phase is Predictive Mean Matching (PMM), a semiparametric approach where missing values are imputed based on a predefined number of observations whose predicted values are closest to the predicted value of the missing case (called donors) (4). From this donor pool one observation is randomly drawn to replace the missing value, ensuring that imputed values are realistic and plausible observations. This method is preferred due to its flexibility of dealing with non-linear relations and its robustness against misspecification of the posterior predictive distribution (4).

All patients characteristic (age, sex), risk factors (diabetes, hypertension, hypercholesterolemia, smoking, BMI) and ECG information (ECG age prediction, heart rhythm) are used to identify the donor pool to maximize the plausibility of the MCAR/MAR assumption. In this study we generated 25 datasets and 5 donor observation for PMM. Convergence of the imputed datasets is confirmed by inspecting the trace plots for stability and absence of any systematic trends in imputed risk factors. Next, during the analysis phase, the desired statistical tests are performed on each imputed dataset separately, resulting in 25 versions of the Chi-squared and ANOVA outcomes. In the pooling phase, results from the multiple analyses get combined to produce a final estimate. For Chi-squared tests, an effective method to pool test statistic is by using the D_2 statistic (5). For the i-th imputed dataset, where $i = 1, \dots, 25$, the combination of Chi-squared statistics d_i is integrated into the D_2 statistic. This statistic transforms the averages test statistic $\bar{d} = \sum_{i=1}^{25} d_i$ by considering the increase of the variance due to missing data. Similarly, the D_1 statistic is used to pool the values for a Wald test of

the ANOVA analysis (6). A large number of imputed datasets are integrated to maximally counteract the decrease in power for imputing test statistics (6).

Supplementary Table 5 presents the missing data pattern across the PAD and PAD_{bc} groups. Modelling the missingness (binary indicator) for smoking and BMI confirms that is statistically significant ($\chi^2 = 7.28$, p< 0.05, $\chi^2 = 10.87$, p< 0.005 for PAD_{bc} and $\chi^2 = 8.75$, p< 0.05, $\chi^2 = 45.37$, p< 0.001 for PAD). This suggests a relationship between the missing data and the variables of interest, implying that mechanisms such as MAR (Missing At Random) or MNAR (Missing Not At Random) may be applicable

For the study, the MAR mechanism is assumed. Under this assumption, the missing data is linked only to observed variables in the dataset and not related to the unobserved variables. More specifically, the missingness in BMI and smoking behaviour may be associated with other recorded risk factors, demographic and ECG information available in the dataset, but may not depend on the information of the missing BMI or smoking data itself. Since BMI and smoking behaviour are highly related to other non-missing variables in the dataset, such as chronological age and comorbidity indications, these relationships are assumed to be sufficient in defining the missing data patterns for both variables. Additionally, after consulting with the clinical staff at Ziekenhuis Oost-Limburg, no explicit mechanism driving the missingness based on the missing variables themselves could be identified. These considerations support the decision to adopt the MAR assumption in the analyses.

Multiple imputations for risk factors analysis

Supplementary Figure 4 and 5 and Supplementary Table 6 and 7 details the imputation process based on PAD and PAD_{bc}, including trace plots and a summary of the final imputed variables. Convergence of the imputation models is achieved as no systematic relationship between the imputed variables can be observed. The stability and lack of systematic trends in the trace plots suggest that the imputation process has converged. The close alignment of the red and blue lines in the density plot indicates that the imputed BMI values closely match the original data distribution. These results ensure the reliability of the imputed values for both PAD and PAD_{bc}.

Both smoking status and BMI remain significantly different across groups after analysis of the imputed datasets. Averaged across all imputed datasets, the presence of smoking was 43.3% for UAD_{bc} (40.7% for UAD), 46.1% for SAD_{bc} (47.1% for SAD), and 48.3% for OAD_{bc} (48.8% for OAD). The variability among imputed datasets was incorporated into the D_2 statistic, combining Chi-squared results. The results of the test indicated significant differences among the groups defined by PAD_{bc} $D_2(2, 20762.24) = 33.11$, $p < 0.001$ ($D_2(2,45722.88) = 102.27$ $p < 0.001$ for PAD). This signifies that smoking behaviour is significantly different across age deviation groups. For imputed BMI variables, the average BMI in the imputed datasets was 26.49 for UAD_{bc} (26.47 for UAD), 27.43 for SAD_{bc} (27.48 for SAD), and 28.16 for OAD_{bc} (28.08 for OAD). The group effect was summarized in the D_1 statistic, with $D_1(2, 8015.56) = 318.97$, $p < 0.001$ ($D_1(2,9424,18) = 312.45$, $p < 0.001$ for PAD), indicating that BMI differs significantly across age deviation groups.

Multiple imputations for the cox model

To avoid bias in the estimation of the coefficients of the Cox proportional hazards model with missing values, the dataset was imputed not only based on patient characteristics (age, sex), risk factors (diabetes, hypertension, hypercholesterolemia, smoking, BMI), and ECG information (ECG predicted age, heart rhythm), but also the mortality indicator and the Nelson-Aalen estimate of the cumulative baseline hazard at survival time. It is advised to use the cumulative hazard instead of the event times or a (logarithmic) transformation thereof to avoid bias coefficient outcomes in the Cox model (7). PMM is again used during the imputation phase. Pooling of the parameter is done using Rubin's rules (8) to produce overall coefficient and variance covariance matrices. The individual iterations can be found in Supplementary Table 12 and 13.

Supplement 3: Model Architecture

The network architecture was previously described in (9) and was implemented in Python using the Keras framework. Training was performed on a distributed Spark cluster with 224 GB random-access memory and 2 Tesla V100 graphics processing unit (NVIDIA).

During training, the following hyperparameters were applied:

- Batch size of 64

- Adam optimizer with an initial learning rate of $3e^{-4}$, which was reduced by a factor 0.1 if the validation loss did not improve for seven consecutive epochs (ReduceLROnPlateau)
- Maximum of 20 epochs with early stopping based on validation performance to prevent overfitting. Training was halted if validation performance did not improve for nine consecutive epochs (EarlyStopping , delta < 0.00001).

The training process ensured that the model saw the entire training set in each epoch, and callbacks (EarlyStopping, and ReduceLROnPlateau) were employed to optimize convergence. The optimal age prediction model converged after 20 epochs.

Supplement 4: Age adjusted Kaplan-Meier

Supplementary Figure 6 shows the age histogram across the different age groups for PAD_{bc} and PAD. These histograms reveal age is distributed equally across PAD_{bc} groups, yet imbalanced for PAD groups, illustrating the age-dependency. Apart from removing the age-bias directly from PAD (PAD_{bc}), the age effect in PAD can be omitted using one of the following two approaches.

1. Evaluation by discretizing age into age bands. (Supplementary Figure 7)
2. Standardizing age distributions across the groups. (Supplementary Figure 8)

By effectively removing the relationship with age, KM curves show better survival in the UAD group compared to the OAD, as was found for the bias corrected PAD_{bc}. These results illustrate the confounding effect of age should be accounted for in age-related analysis, either upfront by adapting the explanatory variable to be age independent or by adjusting for age in the evaluation.

Supplement 5: Cox Proportional Hazards Model Diagnostics and Assumption Checks

Prior to fitting the Cox models, we performed diagnostic checks to ensure validity of key model assumptions. Martingale residual plots revealed a non-linear relationship between chronological age and the log hazard, with a notable inflection around age 50 (Supplementary Figure 9A). Based on model comparison using AIC (Supplementary Table 4), a piecewise linear transformation was applied, treating age effects separately for individuals ≤50 and >50 years. PAD_{bc} did not exhibit a clear visual non-linear relationship (Supplementary Figure 9B), however was further investigated using restricted cubic splines. The functional form revealed a non-linear relationship with a decreasing HR below -10

years in difference with a crossing of HR=1 at -20 years, an increasing trend between -10 years and 10 years and again a decreasing trend above 10 years with a crossing of HR=1 at 25 years (Supplementary Figure 10A). The PAD_{bc}histogram reveals that the bulk of the data is within the -20 to 20 years window supporting an almost linear relationship for most of the data (Supplementary Figure 10B). The authors chose to move forward with the linear approach as this allows for an easier, reader friendly, interpretation of the outcome and PAD_{bc} was modelled without transformation.

For the simple model based on PAD_{bc}, Martingale residuals showed minimal remaining non-linear relationships (Supplementary Figure 11A and 11B). Additionally, Schoenfeld residuals showed no time-dependent bias for PAD_{bc} ($p = 0.92$), supporting the proportional hazards assumption (Supplementary Figure 12A). Though chronological age had a significant Schoenfeld test ($p < 0.001$) (Supplementary Figure 12B), visual inspection did not suggest substantial violation. Log–log survival plots were consistent with proportional hazards (Supplementary Figure 13). Goodness-of-fit was confirmed with Cox-Snell residuals (Supplementary Figure 14), which followed the expected unit exponential distribution and aligned well with the Nelson-Aalen cumulative hazard. Similar findings were obtained for PAD (Supplementary Figures 15–18).
Model assumption for the risk factor Cox proportional hazards model based on PAD_{bc} were performed on the complete case (Supplementary Figure 19 to 21). The results did not indicate any substantial violations. Similar findings were found for the PAD based model, further checks are omitted for conciseness. The complete-case Cox model can be found in Supplementary Table 8 and 9. We further explored the effect of interactions between PAD_{bc} and sex, chronological age and heart rhythm (Supplementary Table 10). For sex, chronological age >50 and rhythm, no significant interactions were found indicating no evidence of effect modification. For chronological age <=50, p=0.008 yet the HR was 0.999 illustrating a minimal effect of interactions with age. Interaction terms were omitted from the final models. Lastly, the individual iterations of the imputations can be found in Supplementary Table 12 and 13. Final estimates were based on the pooled results from imputed datasets, and consistent associations were observed across iterations.

Supplement 6: Supplementary Figure 1: Saliency map depicting the model output gradient with respect to the ECG waveform. Saliency will be large when a small change at the input voltage leads to a large change in age prediction. Dark blue shades indicate regions with the greatest influence on the age prediction. Saliency was averaged over a random sample of 4096 individuals of the main analysis test set. The red waveform depicts the median waveform in each lead among the 4096 individuals.

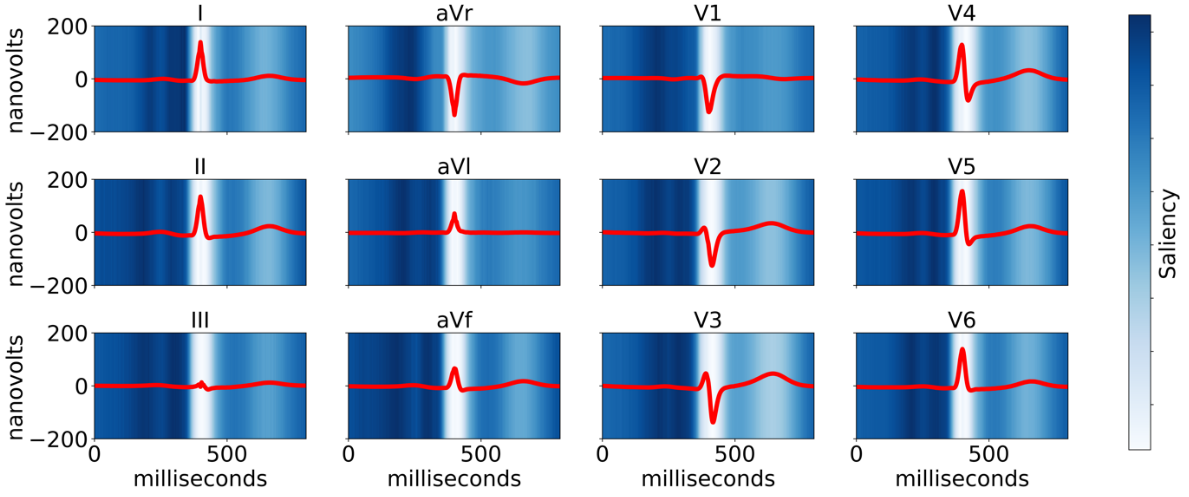

Supplement 7: Supplementary Figure 2: Age histogram for the validation and testset.

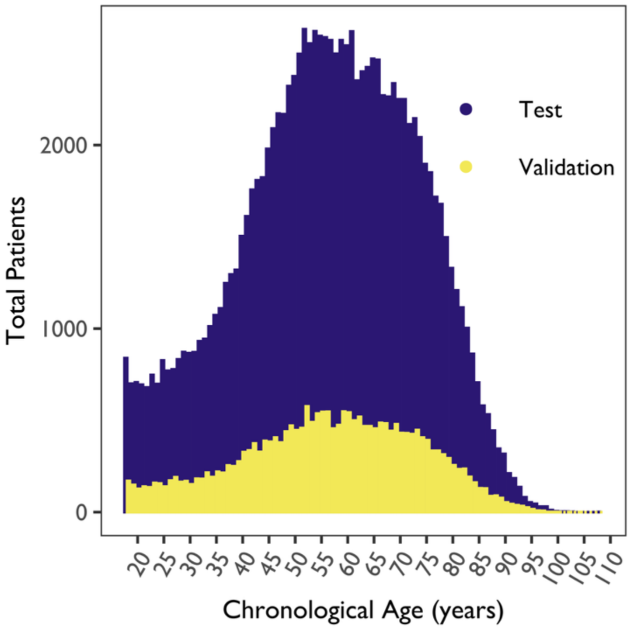

Supplement 8: Supplementary Figure 3: Missing data pattern for all risk factors. (x%) indicates the amount of missing data for this variable.

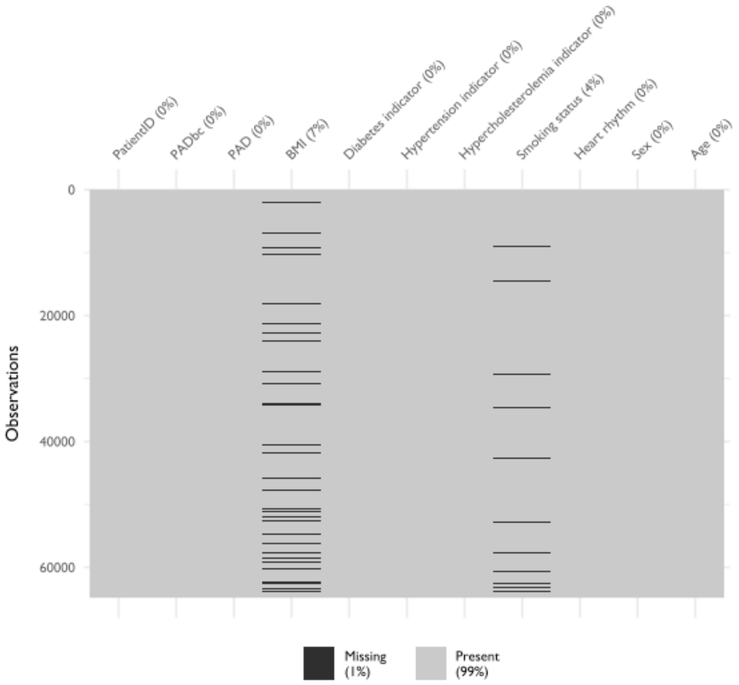

Supplement 9: Supplementary Figure 4: Trace plots for the imputed dataset based on (A) PAD_{bc} and (B) PAD. The trace plots illustrate the convergence diagnostics for the imputed datasets. Each line represents an imputed dataset. $PAD_{(bc)}$, (bias corrected) predicted age deviation.

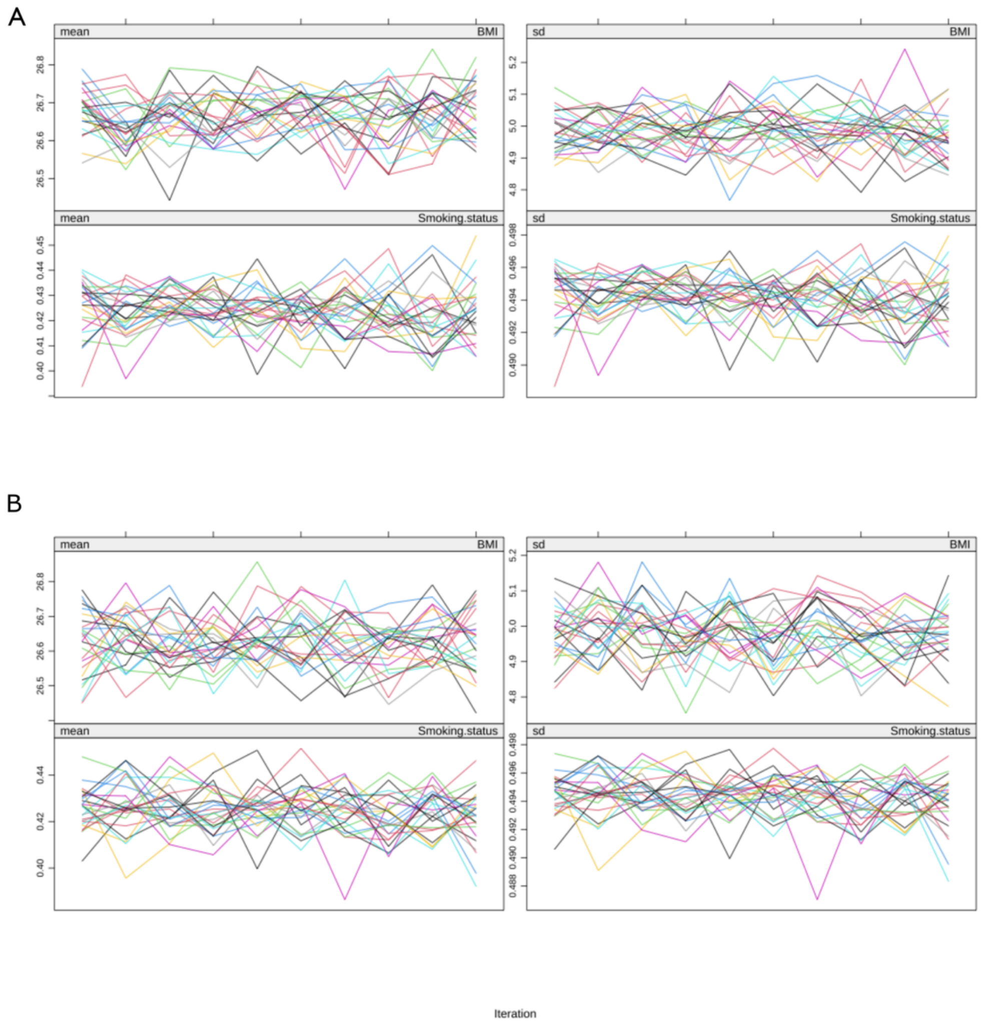

Supplement 10: Supplementary Figure 5: density distribution of BMI values after imputation based on (A) PAD_{bc} and (B) PAD. The original values are shown in blue and the imputed values shown in red. $PAD_{(bc)}$, (bias corrected) predicted age deviation.

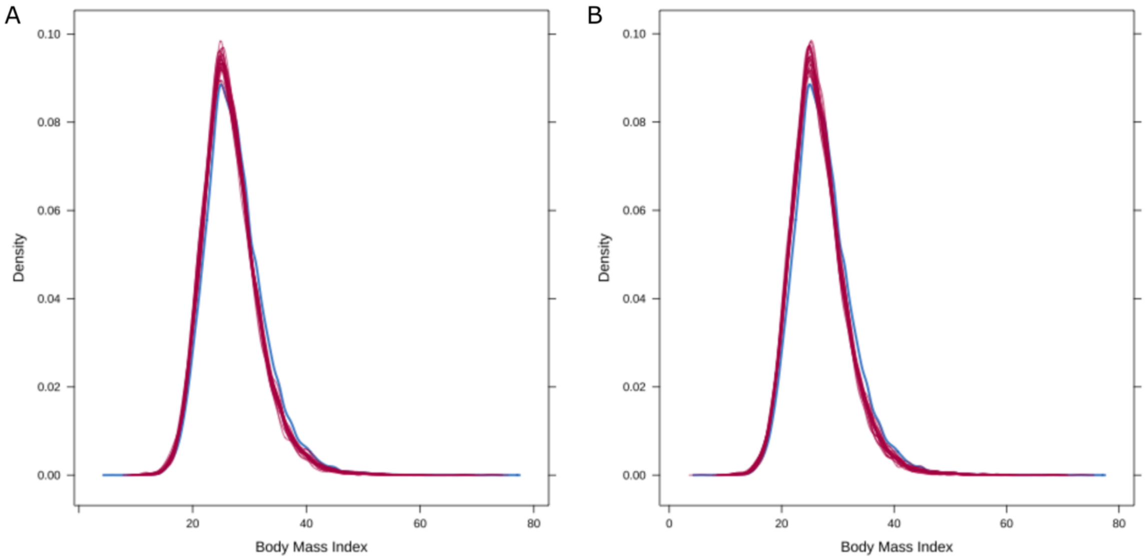

Supplement 11: Supplementary Figure 6: Age histogram for (A) different bias corrected predicted age deviation (PAD_{bc}) groups: UAD_{bc}, SAD_{bc} and OAD_{bc} bias corrected underestimated, small and overestimated age deviation, respectively. (B) different predicted age deviation (PAD) groups: UAD, SAD and OAD underestimated, small and overestimated age deviation, respectively.

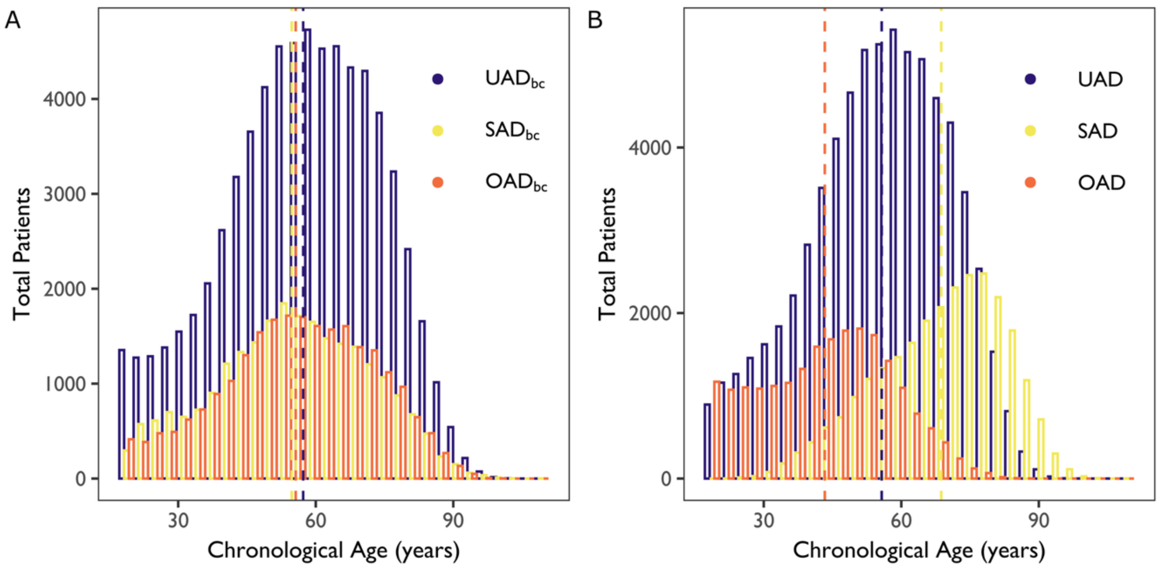

Supplement 12: Supplementary Figure 7: Kaplan-Meier estimates per age band for the uncorrected predicted age deviation (PAD) groups: Underestimated (purple UAD), Small (yellow SAD), and Overestimated age deviation (orange OAD).

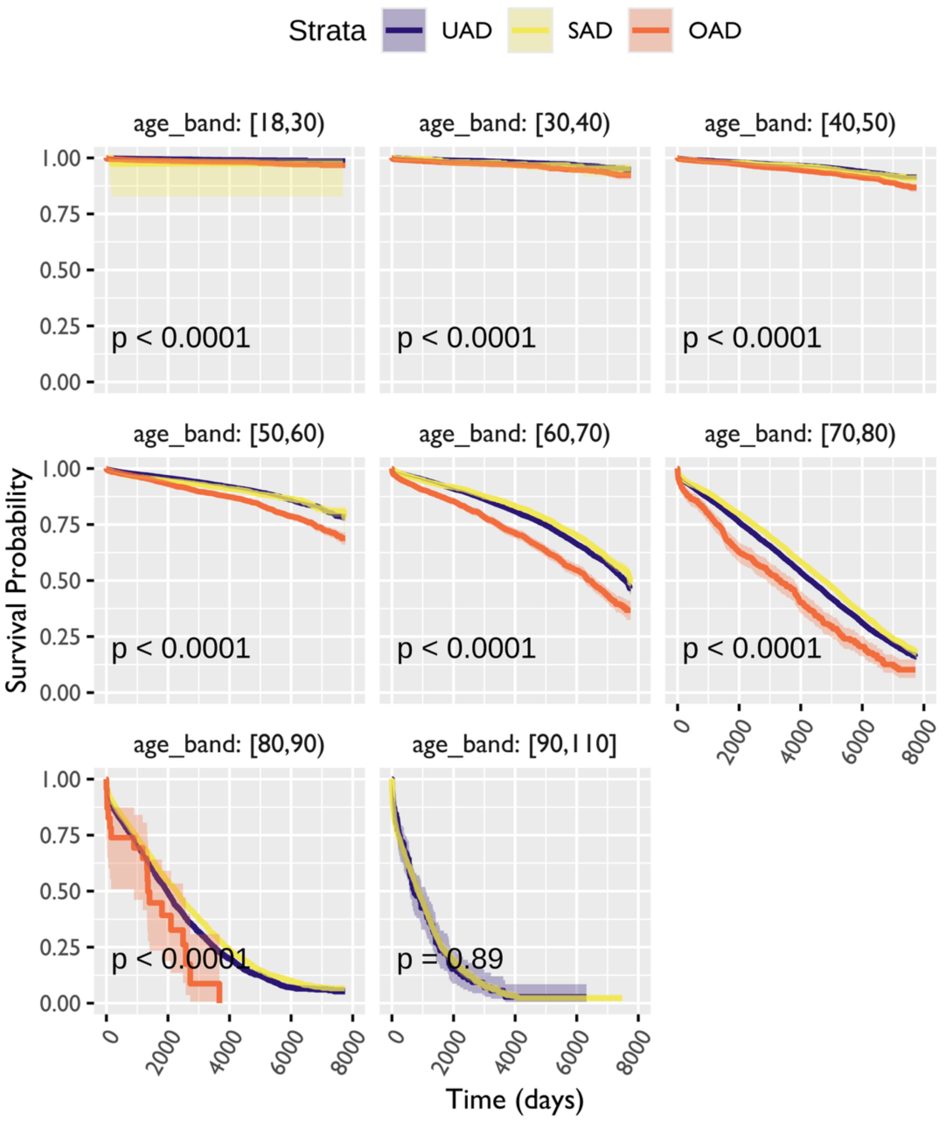

Supplement 13: Supplementary Figure 8: (A) Age histogram and (B) Kaplan-Meier estimates for the age-standardized uncorrected predicted age deviation (PAD) groups: Underestimated (purple UAD), Small (yellow SAD), and Overestimated age deviation (orange OAD).

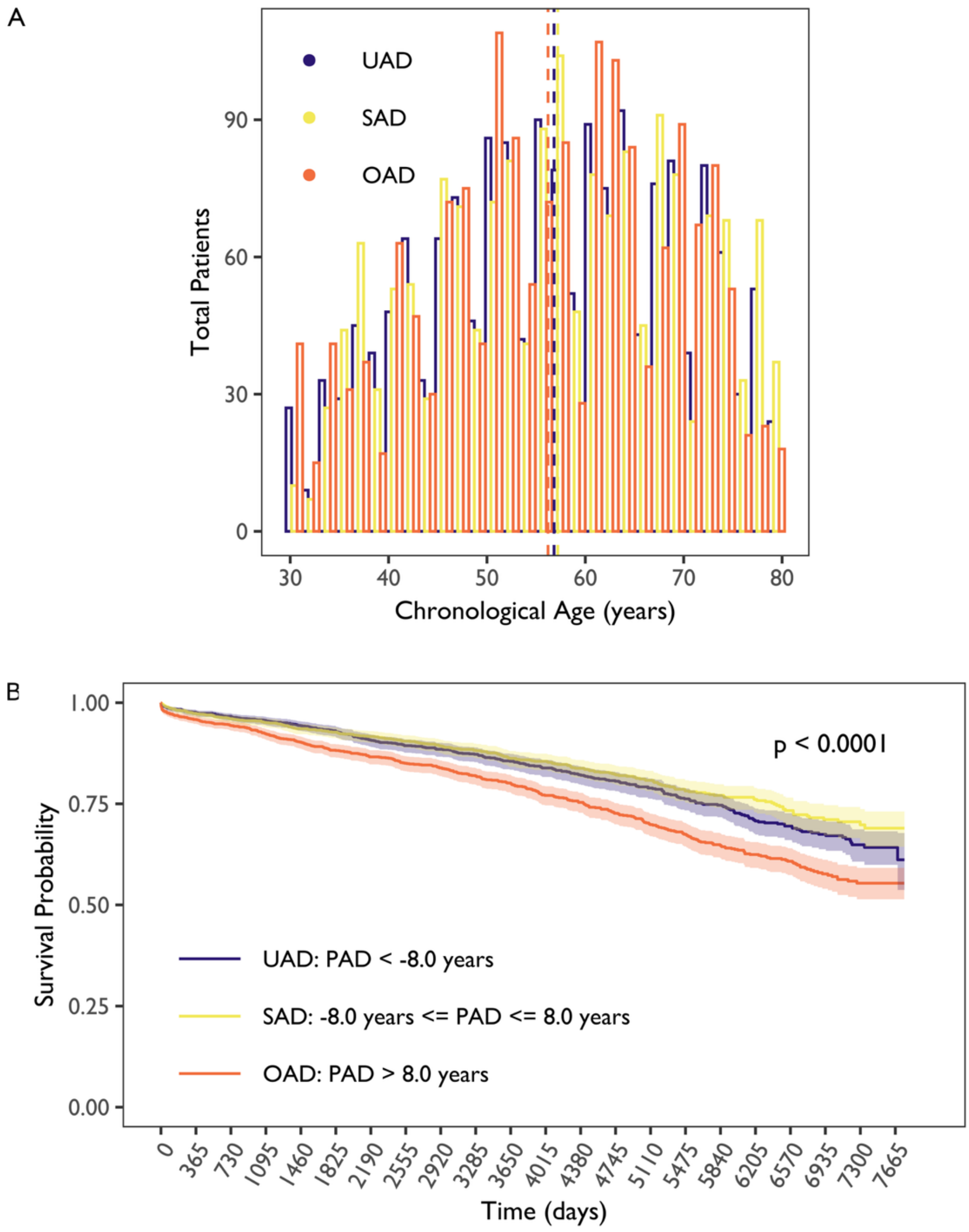

Supplement 14: Supplementary Figure 9: Martingale residuals under the null model for (A) chronological age and (B) under the univariate chronological age-based (piecewise linear) model for the corrected predicted age deviation (PAD_{bc}).

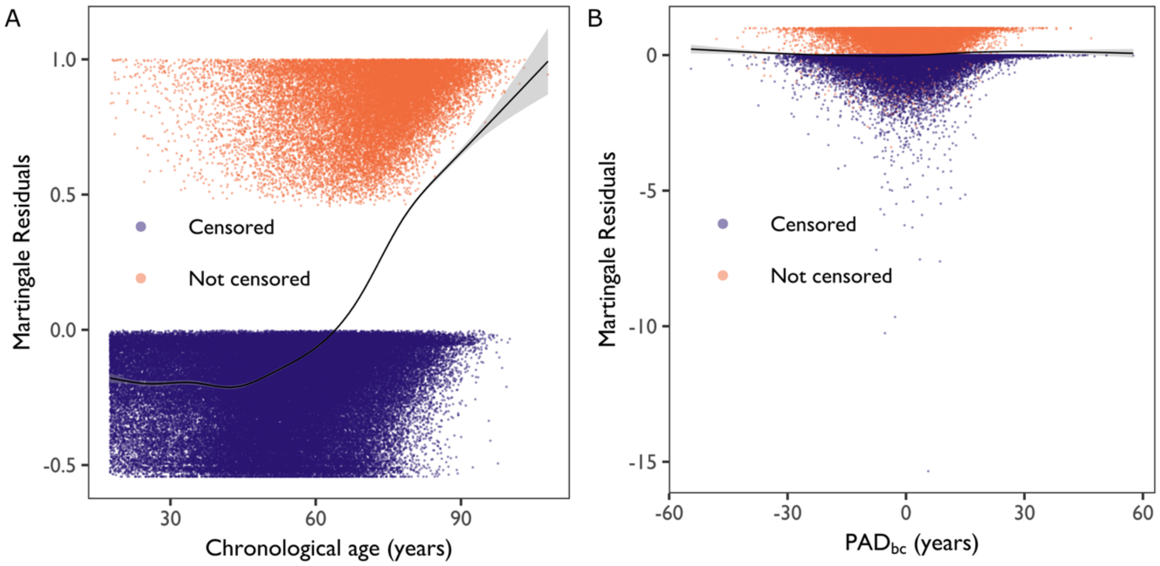

Supplement 15: Supplementary Figure 10: (A) Functional form for the bias corrected predicted age deviation (PAD_{bc}) modelled using restricted cubic splines (B) PAD_{bc} histogram.

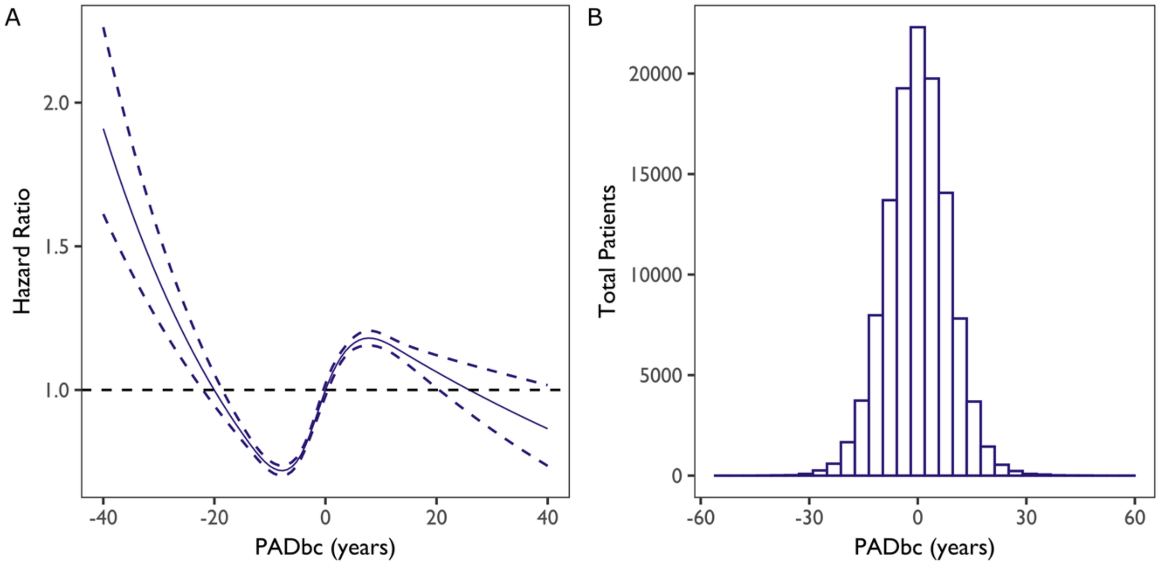

Supplement 16: Supplementary Figure 11: Martingale residuals for (A) chronological age and (B) bias corrected predicted age deviation (PAD_{bc}) after model fit.

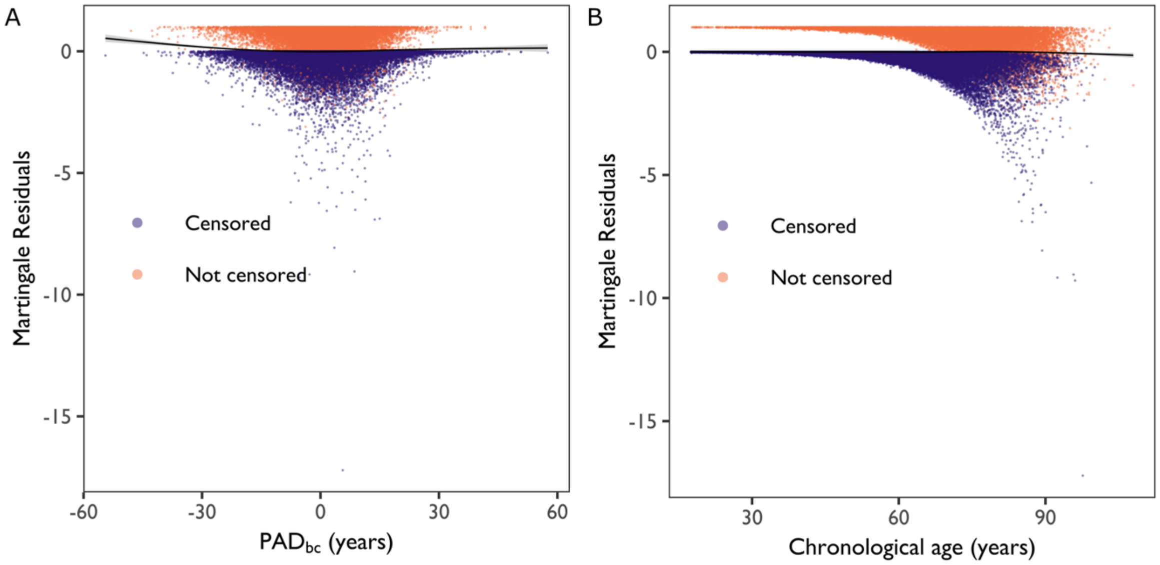

Supplement 17: Supplementary Figure 12: Schoenfeld residual plots for (A) bias corrected predicted age deviation (PAD_{bc}) and (B) chronological age after model fit.

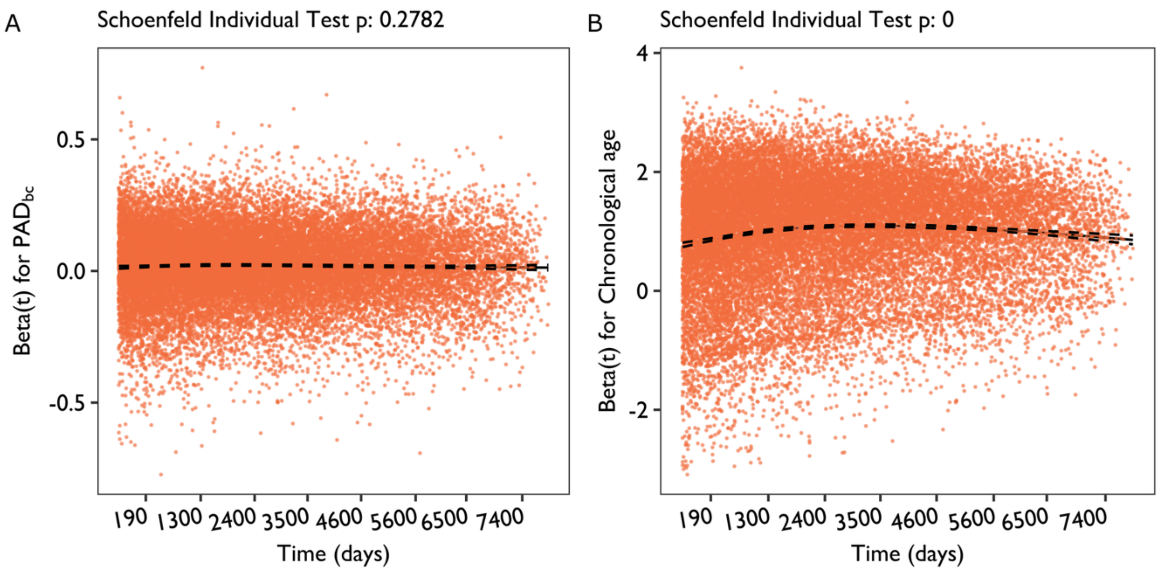

Supplement 18: Supplementary Figure 13: Log-cumulative hazards functions for (A) bias corrected predicted age deviation (PAD_{bc}) and (B) chronological age after model fit.

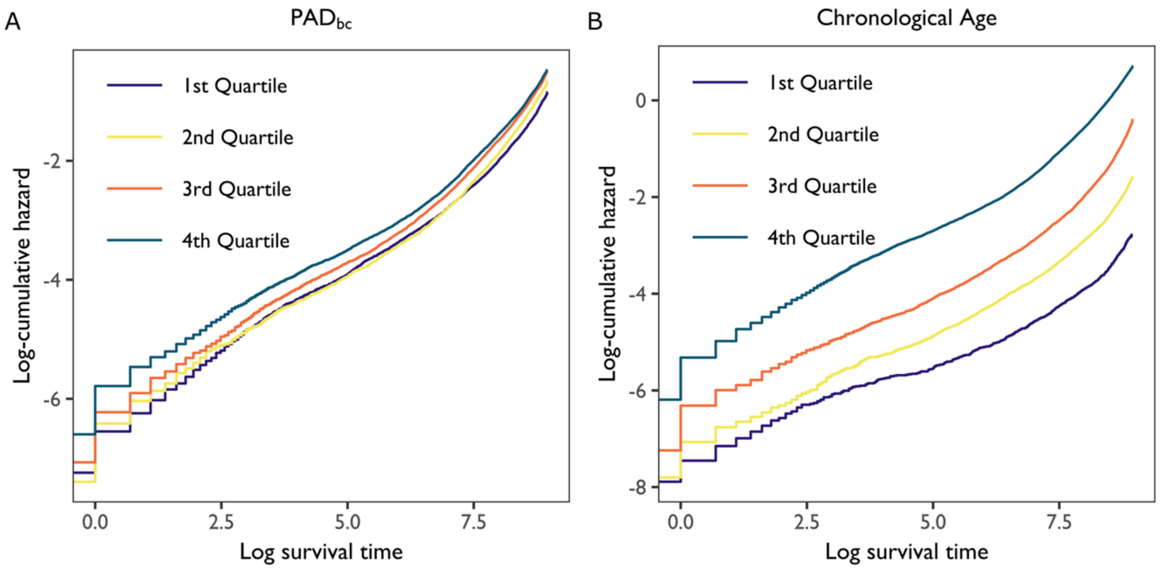

Supplement 19: Supplementary Figure 14: Density distribution of the Cox-Snell residuals for censored observations (histogram) and the Nelson-Aalen estimated cumulative hazard function against the Cox-Snell residuals for the Cox proportional model based on chronological age and the corrected predicted age deviation (PAD_{bc}).

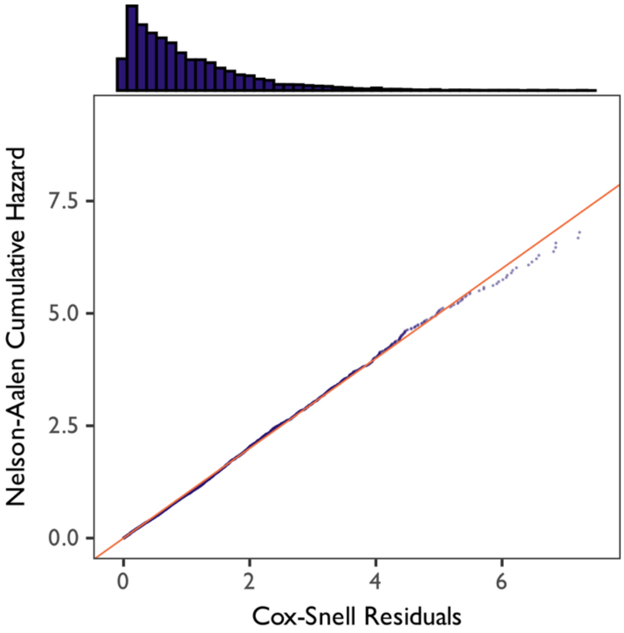

Supplement 20: Supplementary Figure 15: Martingale residuals for (A) predicted age deviation (PAD) and (B) chronological age after model fit.

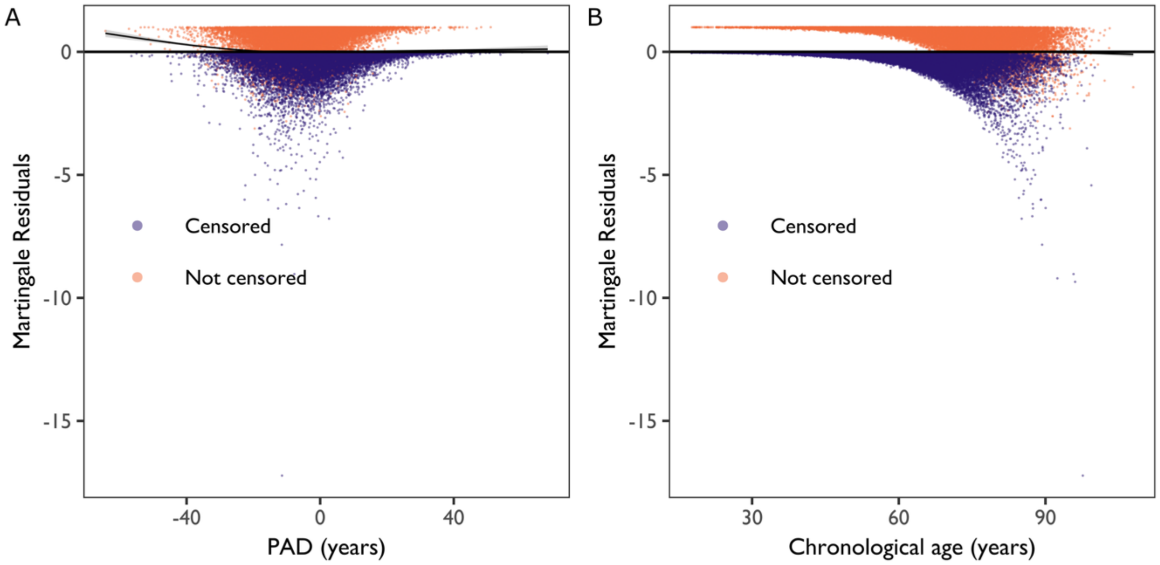

Supplement 21: Supplementary Figure 16: Schoenfeld residual plots for (A) predicted age deviation (PAD) and (B) chronological age after model fit.

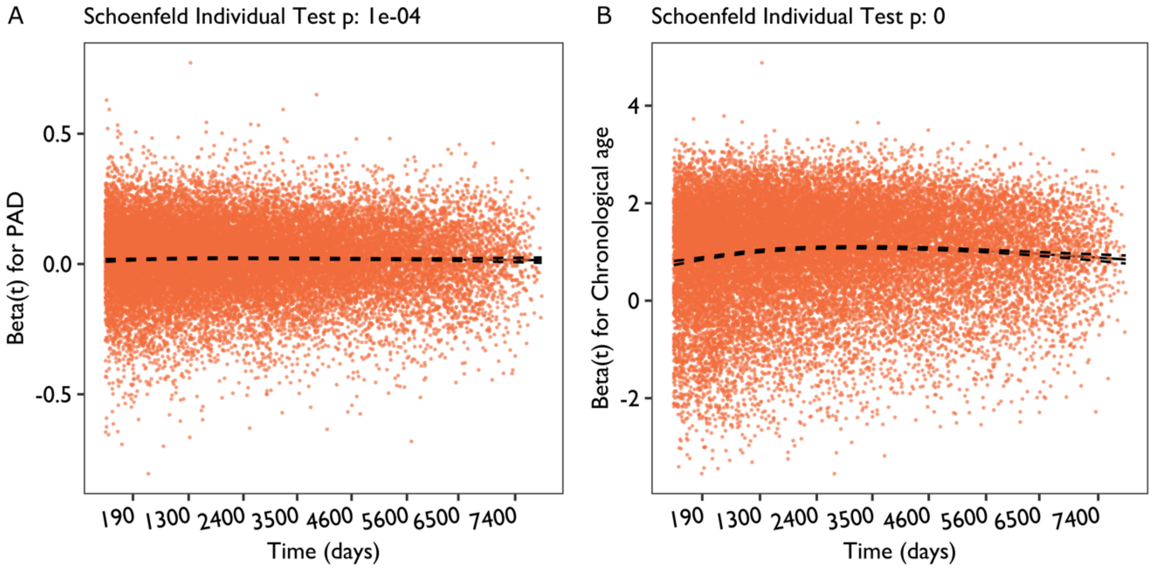

Supplement 22: Supplementary Figure 17: Log-cumulative hazards functions for (A) predicted age deviation (PAD) and (B) chronological age after model fit.

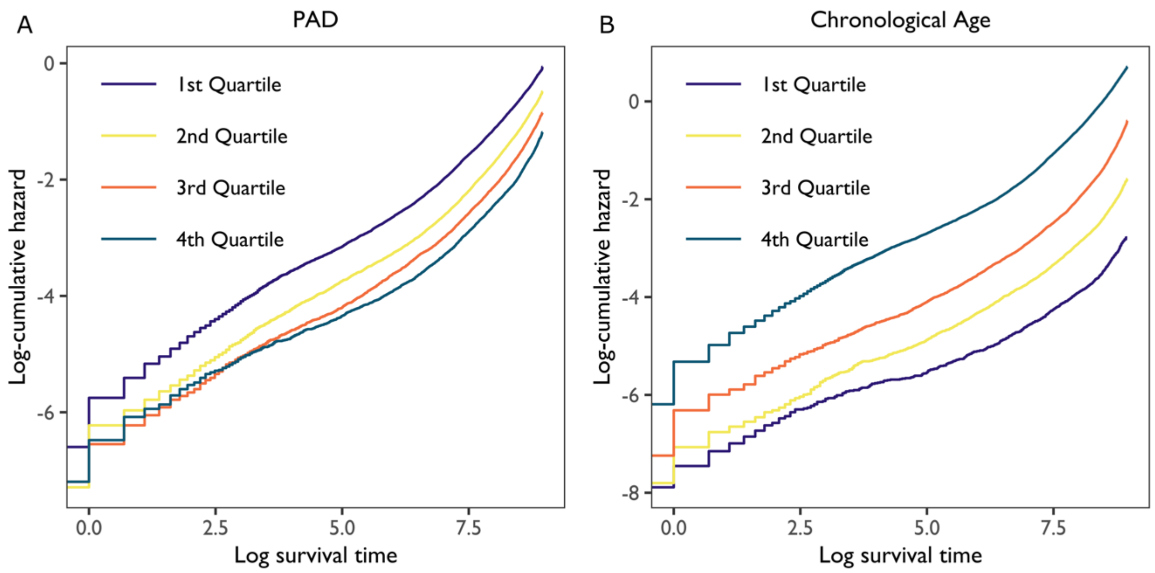

Supplement 23: Supplementary Figure 18: Density distribution of the Cox-Snell residuals for censored observations (histogram) and the Nelson-Aalen estimated cumulative hazard function against the Cox-Snell residuals for the Cox proportional model based on chronological age and predicted age deviation (PAD).

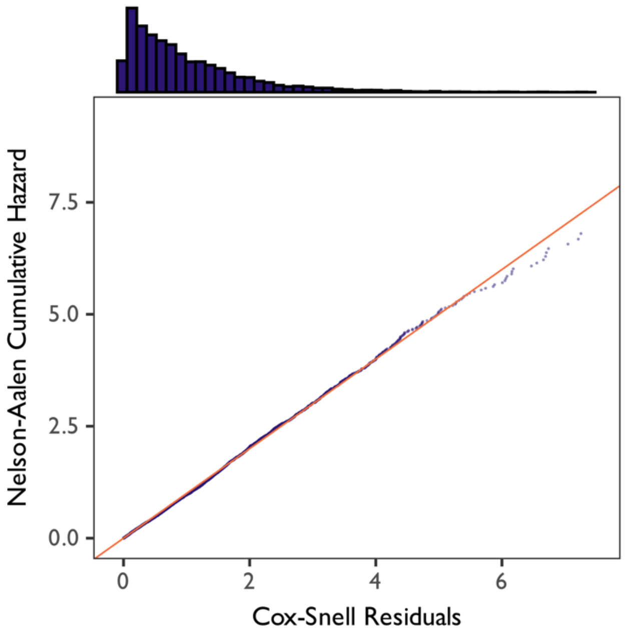

Supplement 24: Supplementary Figure 19: Schoenfeld residual plots for covariates in the risk-adjusted survival model based on the bias corrected predicted age deviation (PAD_{bc}).

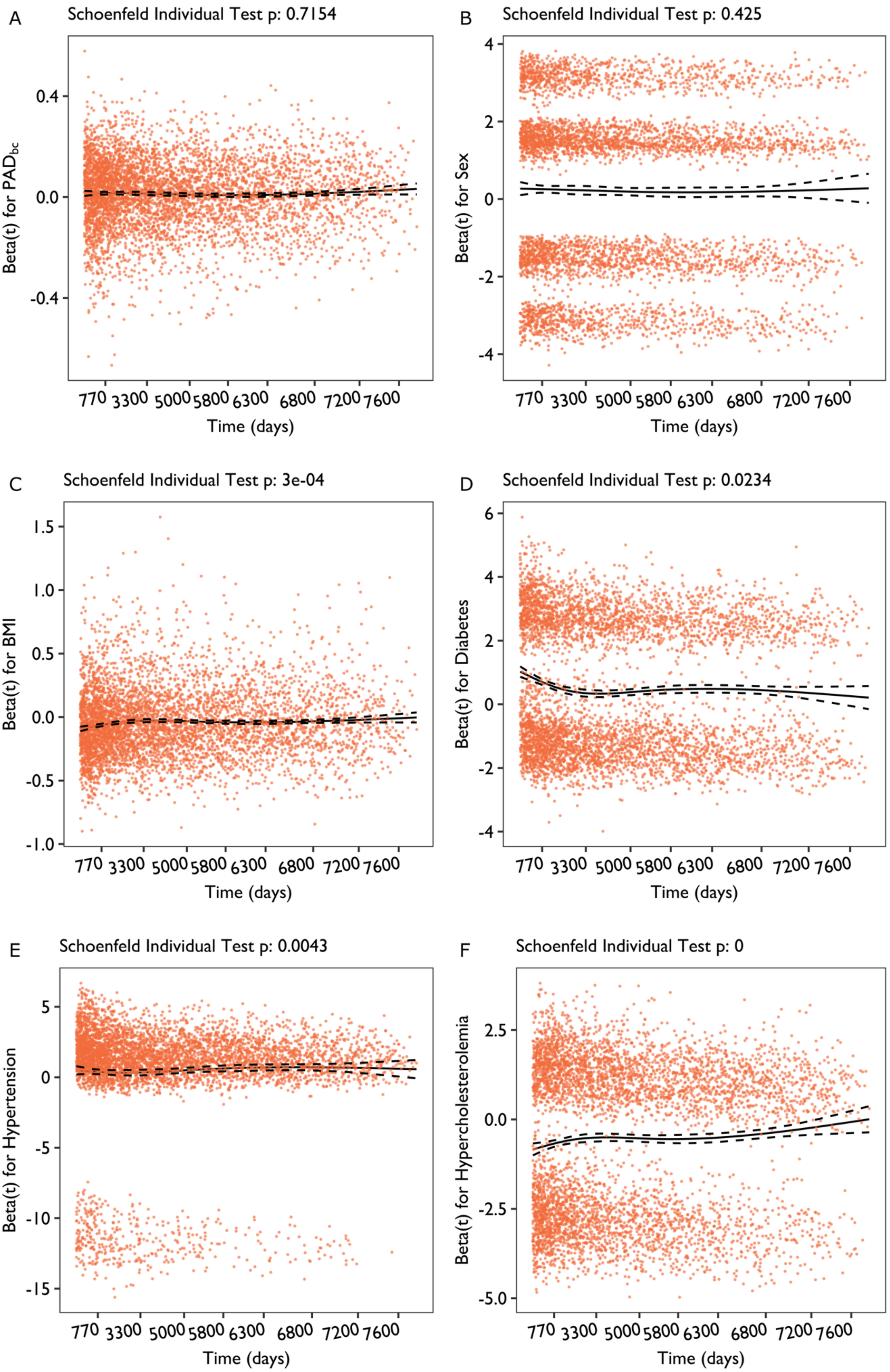
A
Schoenfeld Individual Test p: 0.7154
Beta(t) for PAD_bc
B
Schoenfeld Individual Test p: 0.425
Beta(t) for Sex
C
Schoenfeld Individual Test p: 3e-04
Beta(t) for BMI
D
Schoenfeld Individual Test p: 0.0234
Beta(t) for Diabetes
E
Schoenfeld Individual Test p: 0.0043
Beta(t) for Hypertension
F
Schoenfeld Individual Test p: 0
Beta(t) for Hypercholesterolemia
770 3300 5000 5800 6300 6800 7200 7600
Time (days)

G

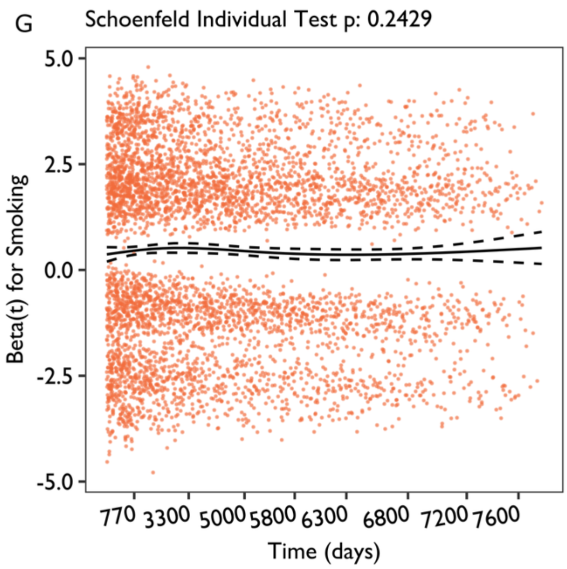
Schoenfeld Individual Test p: 0.2429
Beta(t) for Smoking
5.0
2.5
0.0
-2.5
-5.0
770
3300
5000
5800
6300
6800
7200
7600
Time (days)

H

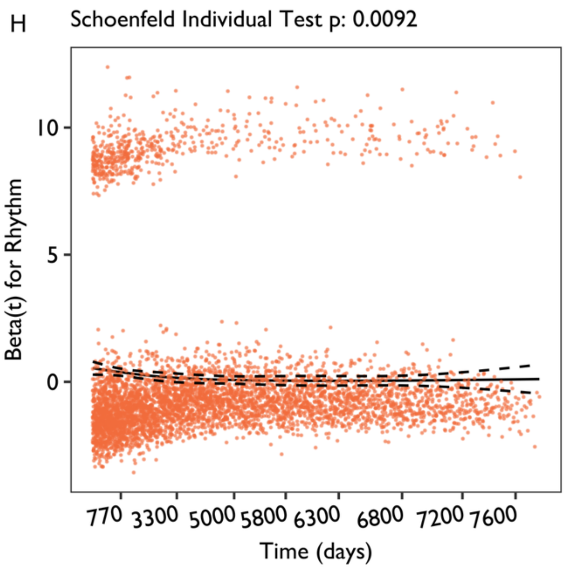
Schoenfeld Individual Test p: 0.0092
Beta(t) for Rhythm
10
5
0
770
3300
5000
5800
6300
6800
7200
7600
Time (days)

I

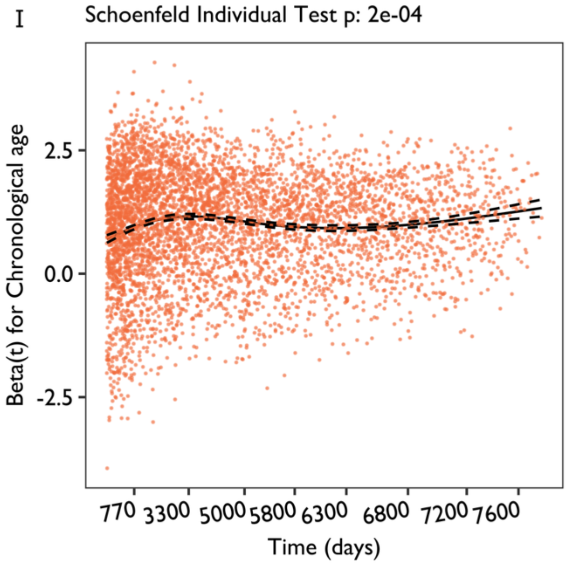
Schoenfeld Individual Test p: 2e-04
Beta(t) for Chronological age
2.5
0.0
-2.5
770
3300
5000
5800
6300
6800
7200
7600
Time (days)

Supplement 25: Supplementary Figure 20: Log-cumulative hazards functions for covariates in the risk-adjusted survival model based on the bias corrected predicted age deviation (PAD_{bc}).

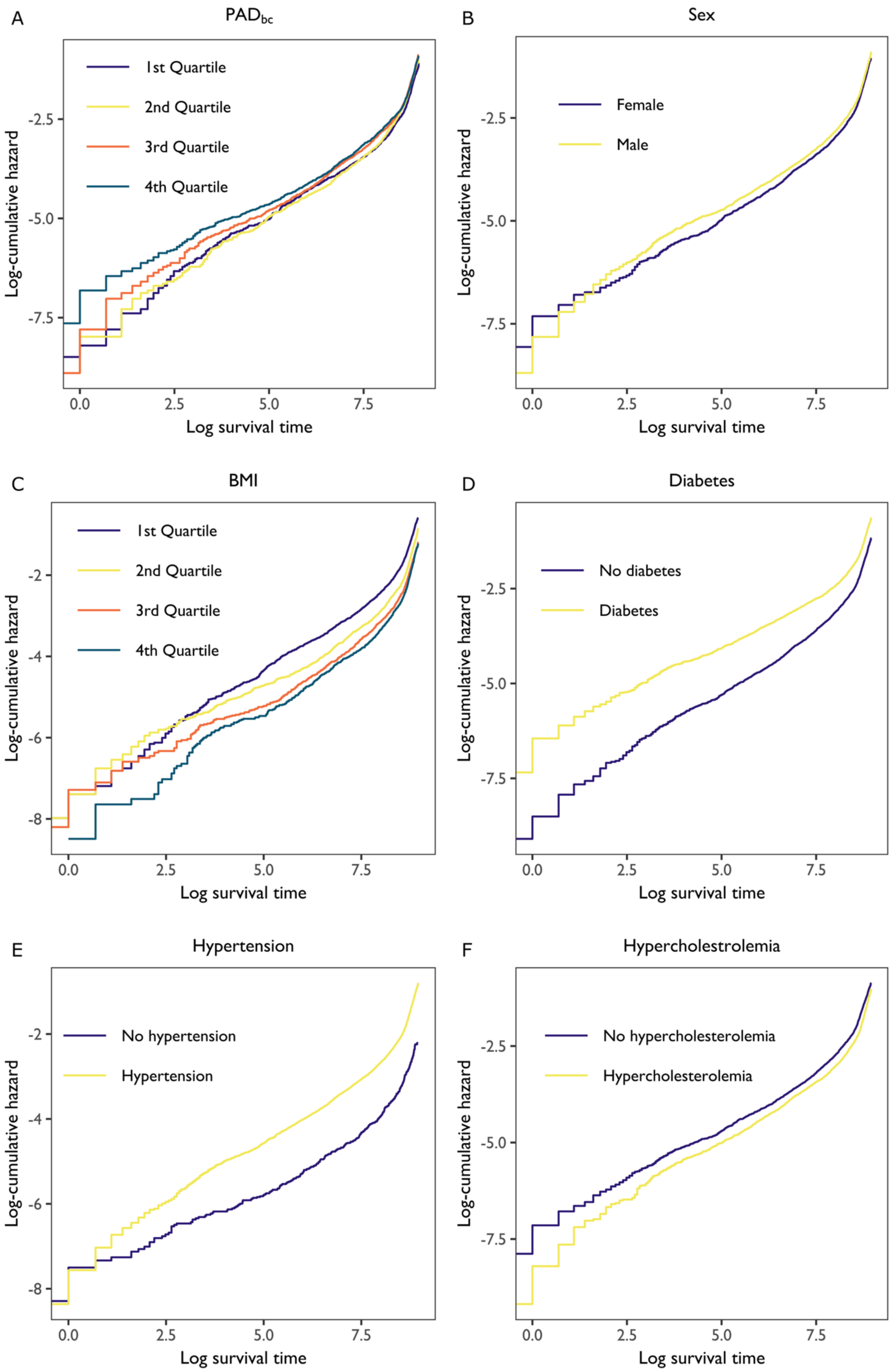
A
PADbc
1st Quartile
2nd Quartile
3rd Quartile
4th Quartile
Log-cumulative hazard
-2.5
-5.0
-7.5
0.0
2.5
5.0
7.5
Log survival time
B
Sex
Female
Male
Log-cumulative hazard
-2.5
-5.0
-7.5
0.0
2.5
5.0
7.5
Log survival time
C
BMI
1st Quartile
2nd Quartile
3rd Quartile
4th Quartile
Log-cumulative hazard
-2
-4
-6
-8
0.0
2.5
5.0
7.5
Log survival time
D
Diabetes
No diabetes
Diabetes
Log-cumulative hazard
-2.5
-5.0
-7.5
0.0
2.5
5.0
7.5
Log survival time
E
Hypertension
No hypertension
Hypertension
Log-cumulative hazard
-2
-4
-6
-8
0.0
2.5
5.0
7.5
Log survival time
F
Hypercholestrolemia
No hypercholesterolemia
Hypercholesterolemia
Log-cumulative hazard
-2.5
-5.0
-7.5
0.0
2.5
5.0
7.5
Log survival time

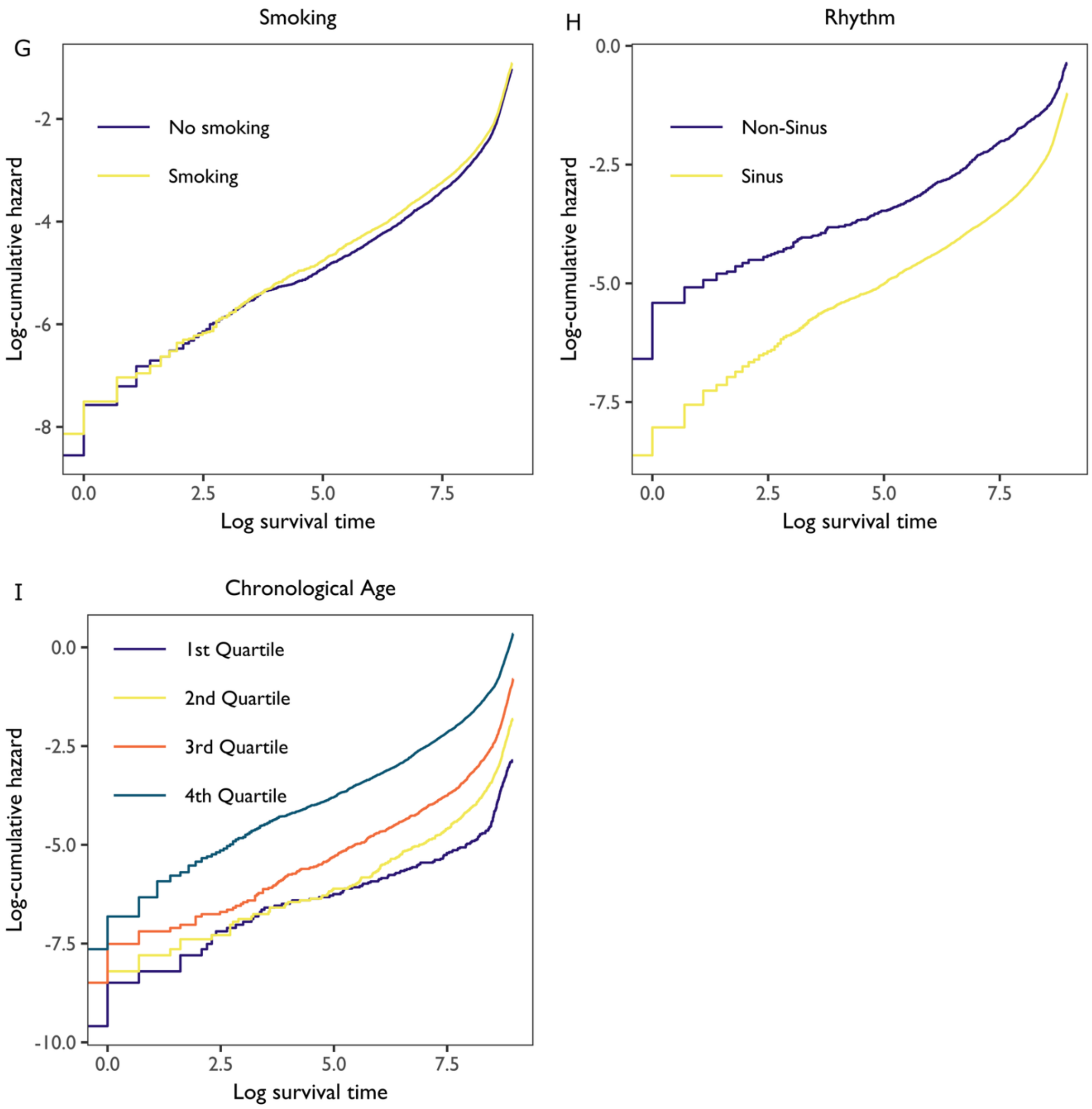

Supplement 26: Supplementary Figure 21: Density distribution of the Cox-Snell residuals for censored observations (histogram) and the Nelson-Aalen estimated cumulative hazard function against the Cox-Snell residuals for the risk-adjusted survival model based on the bias corrected predicted age deviation (PAD_{bc}). The red line represents the expected 45-degree line.

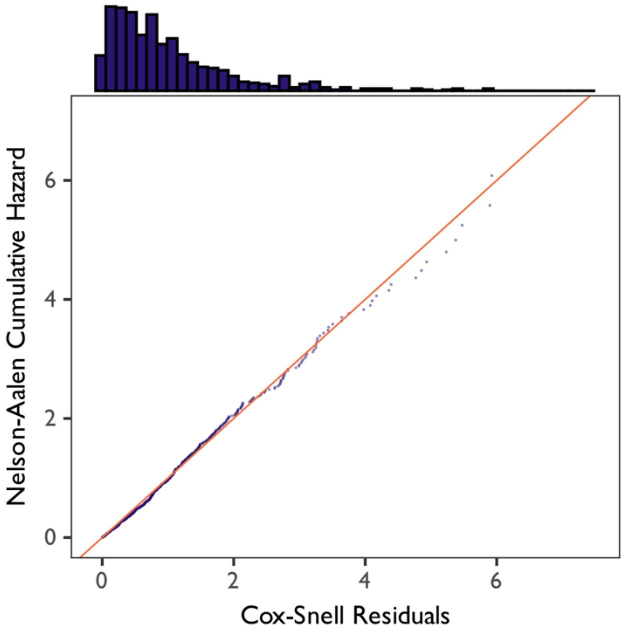

Supplement 27: Supplementary Table 1: Patient characteristics of the model training population (*Age at first ECG).

	Overall	Training set	Validation set	Test set
Patients, n	234,586	93,840	23,378	117,359
ECGs, n	1,258,993	506,003	125,513	627,477
Age*, years	56.9 (±16.0)	56.2 (±16.9)	56.2 (±16.9)	56.4 (±16.9)
Sex, % male	51.6	51.5	51.9	51.7
Atrial fibrillation*, %	3.4	3.4	3.7	3.4
Other Rhythms* %	2.5	2.4	2.5	2.6

Supplement 28: Supplementary Table 2: Patient characteristics of the statistical and survival analysis population (*Age at first ECG).

	General	Risk factors
Patients, n	117,347	64,807
ECGs, n	627,416	215,095

Age*, years	56.4 (±16.9)	60.6 (±16.8)
Sex, % male	51.7	51.1
Atrial fibrillation, %	3.4	4.2
Other Rhythms %	2.6	3.1
Death, %	20.6	8.8
BMI, -	/	27.4 (±5.3) (incomplete data considering 60,067 patients)
Smoking, %	/	45.7 (incomplete data considering 62,809 patients)
Diabetes, %	/	22.4
Hypertension, %	/	62.9
Hypercholesterolemia, %	/	47.2

Supplement 29: Supplementary Table 3: Distribution of cardiovascular risk profile registration vs medication registration for diabetes, hypertension and hypercholesterolemia.

	CV risk profile and Medication	CV risk profile	Medication
Diabetes	14,510	4,532	14,090
Hypertension	40,751	12,921	39,856
Hypercholesterolemia	30,585	14,260	27,317 (26145 statines)

Supplement 30: Supplementary Table 4: Transformations of the chronological age variable and associated AIC of the univariate Cox proportional hazards model.

Chronological age transformation	AIC

Linear chronological age effect	498724.4
Quadratic chronological age effect	498656.9
Interaction with age groups ≤50 and >50	498611.7

Supplement 31: Supplementary Table 5: Percentage of missing data in risk variables (only BMI missing, only smoking missing, and both BMI and smoking missing) in the overall sample and stratified by predicted age deviation (PAD) and bias corrected predicted age deviation (PAD_{bc}) groups. n total number of patients, $UAD_{(bc)}$, $SAD_{(bc)}$ and $OAD_{(bc)}$ (bias corrected) underestimated, small and overestimated age deviation, respectively.

	n	BMI only	Smoking only	BMI and Smoking
Overall	64,807	6.02%	2.55%	1.30%
UAD	14,085	6.18%	2.19%	1.29%
SAD	38,574	5.56%	2.60%	1.28%
OAD	12,148	7.29%	2.81%	1.37%
UAD_{bc}	13,634	6.59%	2.77%	1.37%
SAD_{bc}	38,128	5.79%	2.54%	1.31%
OAD_{bc}	13,045	6.09%	2.34%	1.17%

Supplement 32: Supplementary Table 6: Summary table showing the average BMI and smoking for different bias corrected predicted age deviation (PAD_{bc}) groups across multiple imputations, along with associated chi-squared and F statistics. UAD_{bc}, SAD_{bc} and OAD_{bc} bias corrected underestimated, small and overestimated age deviation, respectively.

Imputation	Variable	UAD_{bc}	SAD_{bc}	OAD_{bc}	χ^2	F
1	% Smoking	43.3%	46.0%	48.4%	70.057	334.598
2		43.3%	46.0%	48.2%	64.500	337.262

3		43.3%	46.1%	48.4%	70.171	336.598
4		43.3%	46.1%	48.3%	68.661	333.118
5		43.1%	46.0%	48.4%	75.778	344.366
6		43.2%	46.0%	48.4%	72.415	337.394
7		43.5%	46.2%	48.5%	67.911	326.521
8		43.2%	46.1%	48.3%	70.424	342.013
9		43.3%	46.1%	48.3%	68.209	342.438
10		43.3%	46.2%	48.3%	69.461	332.149
11		43.3%	46.1%	48.2%	67.123	342.271
12		43.2%	46.2%	48.4%	73.088	334.906
13		43.3%	46.1%	48.2%	64.761	348.654
14		43.3%	46.1%	48.3%	69.078	347.234
15		43.4%	46.2%	48.4%	69.163	346.621
16		43.4%	46.0%	48.2%	62.081	340.289
17		43.5%	46.1%	48.4%	65.608	337.256
18		43.3%	46.1%	48.3%	67.847	346.317
19		43.4%	46.0%	48.4%	66.408	330.527
20		43.3%	46.0%	48.3%	66.974	335.018
21		43.3%	46.0%	48.3%	68.153	348.354
22		43.3%	46.1%	48.5%	71.539	336.613
23		43.3%	46.1%	48.4%	69.454	332.441
24		43.5%	46.1%	48.4%	64.798	337.699
25		43.3%	46.1%	48.4%	69.319	337.734
1	Mean BMI	26.510	27.425	28.165	70.057	334.598
2		26.499	27.430	28.152	64.500	337.262
3		26.507	27.436	28.159	70.171	336.598
4		26.496	27.419	28.137	68.661	333.118
5		26.477	27.424	28.146	75.778	344.366
6		26.488	27.424	28.142	72.415	337.394
7		26.517	27.426	28.152	67.911	326.521
8		26.498	27.426	28.166	70.424	342.013
9		26.482	27.415	28.155	68.209	342.438
10		26.496	27.416	28.142	69.461	332.149
11		26.480	27.424	28.142	67.123	342.271
12		26.494	27.420	28.147	73.088	334.906
13		26.476	27.420	28.161	64.761	348.654

14		26.491	27.423	28.175	69.078	347.234
15		26.485	27.438	28.158	69.163	346.621
16		26.490	27.424	28.151	62.081	340.289
17		26.492	27.425	28.152	65.608	337.256
18		26.488	27.430	28.173	67.847	346.317
19		26.517	27.420	28.162	66.408	330.527
20		26.511	27.426	28.173	66.974	335.018
21		26.476	27.435	28.144	68.153	348.354
22		26.489	27.435	28.134	71.539	336.613
23		26.496	27.427	28.137	69.454	332.441
24		26.510	27.424	28.173	64.798	337.699
25		26.499	27.413	28.167	69.319	337.734

Supplement 33: Supplementary Table 7: Summary table showing the average BMI and smoking for different predicted age deviation (PAD) groups across multiple imputations, along with associated chi-squared and F statistics. UAD, SAD and OAD underestimated, small and overestimated age deviation, respectively.

**Imputation**	**variable**	UAD	SAD	OAD	χ^2	F
1	% Smoking	40.5%	47.1%	48.5%	215.625	346.233
2		40.7%	47.1%	48.6%	210.778	349.816
3		40.7%	47.1%	48.6%	212.229	340.681
4		40.6%	47.0%	48.4%	209.750	350.019
5		40.7%	47.1%	48.6%	209.609	355.312
6		40.7%	47.1%	48.5%	210.358	357.618
7		40.6%	47.2%	48.4%	213.163	342.198
8		40.8%	47.1%	48.4%	202.240	343.553
9		40.7%	47.1%	48.6%	210.024	354.760
10		40.7%	47.0%	48.4%	204.073	349.552
11		40.8%	47.1%	48.5%	205.554	359.876
12		40.8%	47.1%	48.6%	203.620	355.554
13		40.6%	47.1%	48.3%	208.431	353.342
14		40.7%	47.1%	48.6%	209.034	344.543
15		40.7%	47.0%	48.6%	211.915	357.154

16		40.6%	47.1%	48.2%	206.119	353.946
17		40.7%	47.2%	48.4%	209.358	341.681
18		40.6%	47.0%	48.4%	209.578	329.736
19		40.6%	47.1%	48.6%	213.861	336.131
20		40.8%	47.1%	48.4%	197.850	349.321
21		40.7%	47.1%	48.5%	213.093	353.489
22		40.6%	47.1%	48.5%	215.982	336.905
23		40.6%	47.1%	48.5%	211.538	346.397
24		40.7%	47.0%	48.6%	208.938	346.462
25		40.6%	47.0%	48.3%	206.873	358.054
1	Mean BMI	26.466	27.469	28.081	215.625	346.233
2		26.453	27.473	28.068	210.778	349.816
3		26.476	27.472	28.084	212.229	340.681
4		26.467	27.475	28.091	209.750	350.019
5		26.453	27.476	28.086	209.609	355.312
6		26.455	27.487	28.082	210.358	357.618
7		26.473	27.480	28.075	213.163	342.198
8		26.477	27.481	28.085	202.240	343.553
9		26.439	27.472	28.055	210.024	354.760
10		26.468	27.478	28.092	204.073	349.552
11		26.454	27.474	28.102	205.554	359.876
12		26.464	27.489	28.089	203.620	355.554
13		26.471	27.486	28.099	208.431	353.342
14		26.475	27.490	28.076	209.034	344.543
15		26.455	27.481	28.094	211.915	357.154
16		26.460	27.481	28.090	206.119	353.946
17		26.466	27.470	28.059	209.358	341.681
18		26.488	27.474	28.058	209.578	329.736
19		26.475	27.472	28.058	213.861	336.131
20		26.476	27.488	28.096	197.850	349.321
21		26.468	27.491	28.098	213.093	353.489
22		26.492	27.483	28.084	215.982	336.905
23		26.457	27.479	28.058	211.538	346.397
24		26.473	27.480	28.084	208.938	346.462
25		26.449	27.475	28.088	206.873	358.054

Supplement 34: Supplementary Table 8: Complete case Cox proportional hazards model based on chronological age, bias corrected predicted age deviation PAD_{bc} and patient risk factors. HR, Hazard ratio and 95% CI, 95% confidence intervals.

Variable	HR	95% CI	p-value
PAD_{bc}	1.013	1.010, 1.017	<0.001
Sex (M)	1.249	1.175, 1.326	<0.001
BMI	0.956	0.950, 0.962	<0.001
Smoking	1.552	1.460, 1.650	<0.001
Diabetes	1.697	1.600, 1.799	<0.001
Hypertension	1.621	1.458, 1.802	<0.001
Hypercholesterolemia	0.572	0.539, 0.607	<0.001
Heart Rhythm (Non Sinus)	1.224	1.117, 1.341	<0.001
Chronological Age (<=50)	1.058	1.050, 1.067	<0.001
Chronological Age (>50)	1.118	1.114, 1.122	<0.001

Supplement 35: Supplementary Table 9: Complete case Cox proportional hazards model based on chronological age, predicted age deviation (PAD) and patient risk factors. HR, Hazard ratio and 95% CI, 95% confidence intervals.

Variable	HR	95% CI	p-value
PAD	1.013	1.009, 1.016	0,001
Sex (M)	1.248	1.175, 1.326	<0.001
BMI	0.956	0.950, 0.962	<0.001
Smoking	1.552	1.460, 1.650	<0.001
Diabetes	1.697	1.600, 1.799	<0.001
Hypertension	1.623	1.460, 1.805	<0.001
Hypercholesterolemia	0.572	0.539, 0.607	<0.001
Heart Rhythm (Non Sinus)	1.228	1.121, 1.346	<0.001
Chronological Age (<=50)	1.062	1.053, 1.070	<0.001
Chronological Age (>50)	1.124	1.120, 1.129	<0.001

Supplement 36: Supplementary Table 10: Complete case Cox proportional hazards model based on chronological age, bias corrected predicted age deviation (PAD_{bc}) and patient risk factors with interaction terms for age, sex and rhythm. HR, Hazard ratio and 95% CI, 95% confidence intervals

**Variable**	**HR**	**95% CI**	**p-value**
PAD_{bc}	1.010	1.002, 1.017	0.012
Sex (M)	1.246	1.173, 1.324	<0.001
BMI	0.956	0.950, 0.962	<0.001
Smoking	1.551	1.459, 1.649	<0.001
Diabetes	1.697	1.601, 1.799	<0.001
Hypertension	1.618	1.455, 1.799	<0.001
Hypercholesterolemia	0.572	0.439, 0.607	<0.001
Heart Rhythm (Non Sinus)	1.227	1.113, 1.353	<0.001
Chronological Age (<=50)	1.061	1.052, 1.070	<0.001
Chronological Age (>50)	1.118	1.114, 1.122	<0.001
PAD_{bc}: Sex (M)	**1.002**	**0.995, 1.009**	**0.527**
PAD_{bc}: Heart Rhythm (Non Sinus)	**1.000**	**0.990, 1.010**	**0.982**
PAD_{bc}: Chronological Age (<=50)	**0.999**	**0.998, 1.000**	**0.008**
PAD_{bc}: Chronological Age (>50)	**1.000**	**1.000, 1.001**	**0.744**

Supplement 37: Supplementary Table 11: Hazard ratios (HR) for bias corrected predicted age deviation (PAD_{bc}) and hypercholesterolemia comparing different approaches to label hypercholesterolemia within the risk factor model. 95% CI, 95% confidence intervals

**Labelling approach for hypercholesterolemia in risk factor model**	**HR PAD_{bc}**	**HR Hypercholesterolemia**
Registration in cardiovascular risk profile and registration of medication prescription (original definition)	0.013 (p<0.001)	0.572 (p<0.001)
Registration in CV risk profile	1.013 (p<0.001)	0.442 (p<0.001)
Registration in CV risk profile, but no registration of medication prescription	1.013 (p<0.001)	0.487 (p<0.001)

Registration of medication prescription	1.013 (p<0.001)	0.697 (p<0.001)
Registration of medication prescription (split into statins and other medication)	1.013 (p<0.001)	Statins: 0.700 (p<0.001)<br>Other medication: 0.532 (p<0.001)
Registration of medication prescription but no registration in CV risk profile	**1.013 (p<0.002)**	**1.157 (p<0.001)**
Registration of medication prescription (split into statins and other medication) but no registration in CV risk profile	1.013 (p<0.001)	**Statins: 1.170 (p<0.001)**<br>Other medication: 0.885 **(p=0.36)**
Separate variable for registration in CV risk profile and registration of medication prescription	1.013 (p<0.001)	CV risk profile: 0.465 (p<0.001)<br>Medication: 0.808 (p<0.001)
Separate variable for registration in CV risk profile and registration of medication prescription (split into statins and other medication)	1.013 (p<0.001)	CV risk profile: 0.464 (p<0.001)<br>Statins: 0.813 (p<0.001)<br>Other medication: 0.695 (p<0.001)

Supplement 38: Supplementary Table 12: Summary table showing the average Cox proportional models across multiple imputations based on chronological age, bias corrected predicted age deviation PAD_{bc} and patient risk factors. HR, Hazard ratio and 95% CI, 95% confidence intervals.

Iteration	Variable	HR	95% CI	p-value
1		1.014	1.010, 1.017	<0.001
2		1.013	1.010, 1.017	<0.001
3		1.014	1.010, 1.017	<0.001
4		1.013	1.010, 1.017	<0.001
5		1.014	1.010, 1.017	<0.001
6	PAD_{bc}	1.014	1.010, 1.017	<0.001
7		1.014	1.010, 1.017	<0.001
8		1.014	1.010, 1.017	<0.001
9		1.014	1.010, 1.017	<0.001
10		1.014	1.010, 1.017	<0.001
11		1.014	1.010, 1.017	<0.001

12		1.013	1.010, 1.017	<0.001
13		1.014	1.010, 1.017	<0.001
14		1.014	1.010, 1.017	<0.001
15		1.014	1.010, 1.017	<0.001
16		1.014	1.010, 1.017	<0.001
17		1.014	1.010, 1.017	<0.001
18		1.014	1.010, 1.017	<0.001
19		1.014	1.010, 1.017	<0.001
20		1.014	1.010, 1.017	<0.001
21		1.014	1.010, 1.017	<0.001
22		1.014	1.010, 1.017	<0.001
23		1.014	1.010, 1.017	<0.001
24		1.014	1.010, 1.017	<0.001
25		1.014	1.010, 1.017	<0.001
1	Sex (M)	1.266	1.197, 1.340	<0.001
2		1.264	1.195, 1.337	<0.001
3		1.264	1.195, 1.338	<0.001
4		1.267	1.197, 1.340	<0.001
5		1.268	1.199, 1.342	<0.001
6		1.270	1.200, 1.344	<0.001
7		1.266	1.197, 1.340	<0.001
8		1.262	1.193, 1.335	<0.001
9		1.267	1.197, 1.341	<0.001
10		1.266	1.197, 1.340	<0.001
11		1.268	1.199, 1.342	<0.001
12		1.269	1.200, 1.343	<0.001
13		1.269	1.199, 1.342	<0.001
14		1.268	1.198, 1.342	<0.001
15		1.263	1.193, 1.336	<0.001
16		1.265	1.196, 1.339	<0.001
17		1.269	1.199, 1.343	<0.001
18		1.264	1.195, 1.338	<0.001
19		1.264	1.195, 1.338	<0.001
20		1.268	1.198, 1.342	<0.001
21		1.270	1.200, 1.343	<0.001
22		1.271	1.201, 1.344	<0.001

23		1.266	1.197, 1.340	<0.001
24		1.270	1.200, 1.344	<0.001
25		1.264	1.195, 1.337	<0.001
1	BMI	0.954	0.949, 0.960	<0.001
2		0.956	0.951, 0.962	<0.001
3		0.956	0.951, 0.962	<0.001
4		0.956	0.950, 0.961	<0.001
5		0.956	0.950, 0.961	<0.001
6		0.955	0.949, 0.960	<0.001
7		0.957	0.952, 0.963	<0.001
8		0.956	0.950, 0.961	<0.001
9		0.957	0.951, 0.963	<0.001
10		0.956	0.951, 0.962	<0.001
11		0.956	0.950, 0.962	<0.001
12		0.957	0.952, 0.963	<0.001
13		0.956	0.950, 0.962	<0.001
14		0.957	0.952, 0.963	<0.001
15		0.956	0.951, 0.962	<0.001
16		0.957	0.952, 0.963	<0.001
17		0.956	0.951, 0.962	<0.001
18		0.955	0.949, 0.961	<0.001
19		0.957	0.952, 0.963	<0.001
20		0.957	0.951, 0.963	<0.001
21		0.956	0.950, 0.962	<0.001
22		0.957	0.952, 0.963	<0.001
23		0.955	0.950, 0.961	<0.001
24		0.956	0.950, 0.962	<0.001
25		0.956	0.950, 0.961	<0.001
1	Smoking	1.500	1.416, 1.588	<0.001
2		1.493	1.410, 1.581	<0.001
3		1.495	1.412, 1.583	<0.001
4		1.497	1.414, 1.585	<0.001
5		1.488	1.405, 1.575	<0.001
6		1.485	1.402, 1.572	<0.001
7		1.486	1.403, 1.573	<0.001
8		1.508	1.424, 1.597	<0.001

9		1.483	1.401, 1.571	<0.001
10		1.491	1.408, 1.578	<0.001
11		1.483	1.400, 1.570	<0.001
12		1.484	1.402, 1.571	<0.001
13		1.486	1.403, 1.573	<0.001
14		1.488	1.405, 1.576	<0.001
15		1.494	1.411, 1.582	<0.001
16		1.487	1.405, 1.575	<0.001
17		1.491	1.408, 1.579	<0.001
18		1.491	1.408, 1.579	<0.001
19		1.489	1.407, 1.577	<0.001
20		1.482	1.400, 1.569	<0.001
21		1.482	1.399, 1.569	<0.001
22		1.482	1.399, 1.569	<0.001
23		1.497	1.414, 1.585	<0.001
24		1.481	1.398, 1.568	<0.001
25		1.499	1.415, 1.587	<0.001
1	Diabetes	1.688	1.597, 1.784	<0.001
2		1.686	1.595, 1.782	<0.001
3		1.684	1.593, 1.780	<0.001
4		1.683	1.592, 1.779	<0.001
5		1.690	1.599, 1.786	<0.001
6		1.693	1.602, 1.789	<0.001
7		1.689	1.598, 1.785	<0.001
8		1.687	1.596, 1.783	<0.001
9		1.690	1.599, 1.786	<0.001
10		1.690	1.599, 1.786	<0.001
11		1.688	1.597, 1.784	<0.001
12		1.689	1.597, 1.785	<0.001
13		1.690	1.598, 1.786	<0.001
14		1.684	1.593, 1.780	<0.001
15		1.687	1.596, 1.783	<0.001
16		1.686	1.595, 1.782	<0.001
17		1.688	1.597, 1.784	<0.001
18		1.693	1.602, 1.790	<0.001
19		1.687	1.596, 1.783	<0.001

20		1.686	1.595, 1.782	<0.001
21		1.686	1.596, 1.782	<0.001
22		1.684	1.594, 1.780	<0.001
23		1.688	1.597, 1.784	<0.001
24		1.690	1.599, 1.787	<0.001
25		1.690	1.599, 1.786	<0.001
1	Hypertension	1.456	1.332, 1.592	<0.001
2		1.455	1.331, 1.591	<0.001
3		1.452	1.328, 1.587	<0.001
4		1.466	1.341, 1.603	<0.001
5		1.446	1.323, 1.581	<0.001
6		1.449	1.325, 1.584	<0.001
7		1.446	1.323, 1.581	<0.001
8		1.459	1.335, 1.595	<0.001
9		1.439	1.317, 1.574	<0.001
10		1.441	1.318, 1.576	<0.001
11		1.450	1.326, 1.585	<0.001
12		1.451	1.327, 1.586	<0.001
13		1.463	1.338, 1.599	<0.001
14		1.440	1.317, 1.574	<0.001
15		1.453	1.329, 1.589	<0.001
16		1.450	1.326, 1.585	<0.001
17		1.449	1.326, 1.584	<0.001
18		1.456	1.332, 1.592	<0.001
19		1.456	1.331, 1.591	<0.001
20		1.440	1.317, 1.574	<0.001
21		1.442	1.319, 1.576	<0.001
22		1.449	1.325, 1.584	<0.001
23		1.453	1.329, 1.588	<0.001
24		1.449	1.325, 1.584	<0.001
25		1.450	1.326, 1.585	<0.001
1	Hypercholesterolemia	0.573	0.542, 0.606	<0.001
2		0.575	0.544, 0.608	<0.001
3		0.574	0.543, 0.607	<0.001
4		0.575	0.543, 0.608	<0.001
5		0.575	0.543, 0.608	<0.001

6		0.575	0.543, 0.608	<0.001
7		0.574	0.543, 0.607	<0.001
8		0.574	0.543, 0.607	<0.001
9		0.574	0.543, 0.607	<0.001
10		0.576	0.544, 0.609	<0.001
11		0.575	0.544, 0.608	<0.001
12		0.576	0.544, 0.609	<0.001
13		0.575	0.543, 0.608	<0.001
14		0.574	0.543, 0.607	<0.001
15		0.574	0.543, 0.608	<0.001
16		0.575	0.543, 0.608	<0.001
17		0.576	0.545, 0.609	<0.001
18		0.574	0.543, 0.607	<0.001
19		0.574	0.542, 0.607	<0.001
20		0.575	0.543, 0.608	<0.001
21		0.574	0.543, 0.607	<0.001
22		0.574	0.543, 0.607	<0.001
23		0.575	0.543, 0.608	<0.001
24		0.574	0.543, 0.607	<0.001
25		0.574	0.542, 0.607	<0.001
1		1.252	1.151, 1.362	<0.001
2		1.264	1.163, 1.375	<0.001
3		1.259	1.158, 1.370	<0.001
4		1.260	1.158, 1.370	<0.001
5		1.259	1.158, 1.369	<0.001
6		1.260	1.158, 1.370	<0.001
7		1.264	1.162, 1.374	<0.001
8		1.254	1.153, 1.364	<0.001
9	Heart Rhythm (Non Sinus)	1.260	1.158, 1.370	<0.001
10		1.269	1.167, 1.380	<0.001
11		1.260	1.159, 1.371	<0.001
12		1.258	1.157, 1.368	<0.001
13		1.256	1.155, 1.366	<0.001
14		1.261	1.159, 1.371	<0.001
15		1.259	1.158, 1.369	<0.001
16		1.262	1.161, 1.372	<0.001

17		1.259	1.157, 1.369	<0.001
18		1.259	1.157, 1.369	<0.001
19		1.254	1.153, 1.364	<0.001
20		1.256	1.155, 1.366	<0.001
21		1.259	1.158, 1.369	<0.001
22		1.254	1.153, 1.364	<0.001
23		1.258	1.157, 1.368	<0.001
24		1.257	1.156, 1.367	<0.001
25		1.253	1.152, 1.362	<0.001
1	Chronological Age (<=50)	1.051	1.043, 1.059	<0.001
2		1.051	1.043, 1.059	<0.001
3		1.051	1.043, 1.059	<0.001
4		1.051	1.043, 1.059	<0.001
5		1.051	1.043, 1.059	<0.001
6		1.051	1.043, 1.059	<0.001
7		1.051	1.043, 1.059	<0.001
8		1.051	1.043, 1.059	<0.001
9		1.051	1.043, 1.059	<0.001
10		1.051	1.043, 1.059	<0.001
11		1.051	1.043, 1.059	<0.001
12		1.051	1.043, 1.059	<0.001
13		1.051	1.043, 1.059	<0.001
14		1.051	1.043, 1.059	<0.001
15		1.051	1.043, 1.059	<0.001
16		1.051	1.043, 1.059	<0.001
17		1.051	1.043, 1.059	<0.001
18		1.051	1.043, 1.059	<0.001
19		1.051	1.043, 1.059	<0.001
20		1.051	1.043, 1.059	<0.001
21		1.051	1.043, 1.059	<0.001
22		1.051	1.043, 1.059	<0.001
23		1.051	1.043, 1.059	<0.001
24		1.051	1.043, 1.059	<0.001
25		1.051	1.043, 1.059	<0.001
1	Chronological Age (>50)	1.120	1.116, 1.124	<0.001
2		1.120	1.116, 1.124	<0.001

3		1.120	1.116, 1.124	<0.001
4		1.120	1.116, 1.124	<0.001
5		1.120	1.116, 1.124	<0.001
6		1.120	1.116, 1.124	<0.001
7		1.120	1.116, 1.124	<0.001
8		1.120	1.116, 1.124	<0.001
9		1.120	1.117, 1.124	<0.001
10		1.120	1.117, 1.124	<0.001
11		1.120	1.116, 1.124	<0.001
12		1.120	1.116, 1.124	<0.001
13		1.120	1.116, 1.124	<0.001
14		1.121	1.117, 1.124	<0.001
15		1.120	1.116, 1.124	<0.001
16		1.120	1.117, 1.124	<0.001
17		1.120	1.116, 1.124	<0.001
18		1.120	1.116, 1.124	<0.001
19		1.120	1.117, 1.124	<0.001
20		1.120	1.117, 1.124	<0.001
21		1.120	1.116, 1.124	<0.001
22		1.120	1.116, 1.124	<0.001
23		1.120	1.116, 1.124	<0.001
24		1.120	1.116, 1.124	<0.001
25		1.120	1.117, 1.124	<0.001

Supplement 39: Supplementary Table 13: Summary table showing the average Cox proportional models across multiple imputations based on chronological age, predicted age deviation (PAD) and patient risk factors. HR, Hazard ratio and 95% CI, 95% confidence intervals.

Iteration	Variable	HR	95% CI	p-value
1	PAD	1.013	1.010, 1.016	<0.001
2		1.013	1.010, 1.016	<0.001
3		1.013	1.009, 1.016	<0.001
4		1.013	1.009, 1.016	<0.001

5		1.013	1.010, 1.016	<0.001
6		1.013	1.010, 1.016	<0.001
7		1.013	1.009, 1.016	<0.001
8		1.013	1.010, 1.016	<0.001
9		1.013	1.010, 1.016	<0.001
10		1.013	1.009, 1.016	<0.001
11		1.013	1.009, 1.016	<0.001
12		1.013	1.009, 1.016	<0.001
13		1.013	1.009, 1.016	<0.001
14		1.013	1.009, 1.016	<0.001
15		1.013	1.010, 1.016	<0.001
16		1.013	1.009, 1.016	<0.001
17		1.013	1.010, 1.016	<0.001
18		1.013	1.009, 1.016	<0.001
19		1.013	1.009, 1.016	<0.001
20		1.013	1.009, 1.016	<0.001
21		1.013	1.010, 1.016	<0.001
22		1.013	1.009, 1.016	<0.001
23		1.013	1.010, 1.016	<0.001
24		1.013	1.009, 1.016	<0.001
25		1.013	1.009, 1.016	<0.001
1	Sex (M)	1.262	1.193, 1.335	<0.001
2		1.263	1.194, 1.337	<0.001
3		1.262	1.192, 1.335	<0.001
4		1.258	1.189, 1.331	<0.001
5		1.266	1.196, 1.339	<0.001
6		1.267	1.197, 1.340	<0.001
7		1.266	1.196, 1.339	<0.001
8		1.263	1.194, 1.336	<0.001
9		1.269	1.200, 1.343	<0.001
10		1.267	1.197, 1.340	<0.001
11		1.270	1.200, 1.344	<0.001
12		1.271	1.201, 1.345	<0.001
13		1.265	1.196, 1.339	<0.001
14		1.265	1.196, 1.339	<0.001
15		1.263	1.193, 1.336	<0.001

16		1.266	1.197, 1.340	<0.001
17		1.265	1.195, 1.338	<0.001
18		1.264	1.194, 1.337	<0.001
19		1.265	1.195, 1.338	<0.001
20		1.265	1.195, 1.339	<0.001
21		1.273	1.204, 1.347	<0.001
22		1.266	1.197, 1.340	<0.001
23		1.268	1.198, 1.342	<0.001
24		1.269	1.199, 1.342	<0.001
25		1.264	1.195, 1.338	<0.001
1	BMI	0.955	0.949, 0.960	<0.001
2		0.954	0.948, 0.959	<0.001
3		0.957	0.952, 0.963	<0.001
4		0.956	0.950, 0.961	<0.001
5		0.956	0.950, 0.961	<0.001
6		0.954	0.949, 0.960	<0.001
7		0.957	0.951, 0.962	<0.001
8		0.954	0.948, 0.959	<0.001
9		0.955	0.949, 0.960	<0.001
10		0.955	0.949, 0.960	<0.001
11		0.956	0.950, 0.961	<0.001
12		0.957	0.951, 0.962	<0.001
13		0.958	0.952, 0.963	<0.001
14		0.958	0.952, 0.963	<0.001
15		0.955	0.949, 0.960	<0.001
16		0.955	0.950, 0.961	<0.001
17		0.954	0.949, 0.960	<0.001
18		0.956	0.950, 0.961	<0.001
19		0.956	0.951, 0.962	<0.001
20		0.955	0.949, 0.961	<0.001
21		0.953	0.948, 0.959	<0.001
22		0.958	0.952, 0.963	<0.001
23		0.956	0.950, 0.961	<0.001
24		0.955	0.949, 0.960	<0.001
25		0.957	0.951, 0.962	<0.001
1	Smoking	1.499	1.415, 1.587	<0.001

2		1.496	1.413, 1.584	<0.001
3		1.494	1.411, 1.582	<0.001
4		1.500	1.417, 1.588	<0.001
5		1.486	1.403, 1.573	<0.001
6		1.487	1.404, 1.574	<0.001
7		1.488	1.405, 1.576	<0.001
8		1.506	1.423, 1.595	<0.001
9		1.482	1.400, 1.569	<0.001
10		1.489	1.406, 1.577	<0.001
11		1.485	1.402, 1.572	<0.001
12		1.483	1.401, 1.570	<0.001
13		1.486	1.404, 1.574	<0.001
14		1.488	1.406, 1.576	<0.001
15		1.497	1.413, 1.585	<0.001
16		1.491	1.408, 1.578	<0.001
17		1.495	1.412, 1.583	<0.001
18		1.494	1.411, 1.582	<0.001
19		1.491	1.408, 1.579	<0.001
20		1.492	1.409, 1.580	<0.001
21		1.472	1.390, 1.558	<0.001
22		1.483	1.400, 1.570	<0.001
23		1.500	1.417, 1.588	<0.001
24		1.479	1.396, 1.565	<0.001
25		1.498	1.415, 1.586	<0.001
1	Diabetes	1.691	1.600, 1.788	<0.001
2		1.699	1.607, 1.795	<0.001
3		1.690	1.599, 1.786	<0.001
4		1.687	1.596, 1.783	<0.001
5		1.690	1.599, 1.786	<0.001
6		1.693	1.602, 1.789	<0.001
7		1.686	1.596, 1.783	<0.001
8		1.696	1.604, 1.792	<0.001
9		1.692	1.601, 1.788	<0.001
10		1.690	1.599, 1.786	<0.001
11		1.688	1.597, 1.785	<0.001
12		1.690	1.599, 1.787	<0.001

13		1.682	1.591, 1.777	<0.001
14		1.683	1.593, 1.779	<0.001
15		1.691	1.600, 1.788	<0.001
16		1.686	1.595, 1.782	<0.001
17		1.690	1.599, 1.786	<0.001
18		1.693	1.601, 1.789	<0.001
19		1.689	1.598, 1.785	<0.001
20		1.691	1.600, 1.788	<0.001
21		1.698	1.607, 1.795	<0.001
22		1.681	1.591, 1.777	<0.001
23		1.690	1.599, 1.786	<0.001
24		1.696	1.604, 1.792	<0.001
25		1.694	1.602, 1.790	<0.001
1	Hypertension	1.440	1.317, 1.574	<0.001
2		1.451	1.327, 1.586	<0.001
3		1.454	1.330, 1.590	<0.001
4		1.458	1.333, 1.594	<0.001
5		1.442	1.319, 1.577	<0.001
6		1.449	1.325, 1.584	<0.001
7		1.453	1.329, 1.589	<0.001
8		1.467	1.341, 1.603	<0.001
9		1.444	1.321, 1.579	<0.001
10		1.463	1.338, 1.599	<0.001
11		1.451	1.328, 1.587	<0.001
12		1.455	1.331, 1.591	<0.001
13		1.450	1.326, 1.585	<0.001
14		1.442	1.319, 1.576	<0.001
15		1.457	1.333, 1.593	<0.001
16		1.454	1.330, 1.590	<0.001
17		1.458	1.334, 1.594	<0.001
18		1.451	1.327, 1.586	<0.001
19		1.452	1.328, 1.588	<0.001
20		1.451	1.328, 1.587	<0.001
21		1.453	1.329, 1.588	<0.001
22		1.454	1.330, 1.590	<0.001
23		1.457	1.333, 1.593	<0.001

24		1.456	1.332, 1.592	<0.001
25		1.451	1.327, 1.586	<0.001
1	Hypercholesterolemia	0.575	0.544, 0.608	<0.001
2		0.577	0.545, 0.610	<0.001
3		0.574	0.542, 0.607	<0.001
4		0.574	0.543, 0.607	<0.001
5		0.576	0.545, 0.610	<0.001
6		0.576	0.545, 0.610	<0.001
7		0.574	0.543, 0.607	<0.001
8		0.574	0.543, 0.607	<0.001
9		0.575	0.544, 0.608	<0.001
10		0.575	0.544, 0.608	<0.001
11		0.576	0.544, 0.609	<0.001
12		0.574	0.543, 0.607	<0.001
13		0.573	0.542, 0.606	<0.001
14		0.574	0.543, 0.608	<0.001
15		0.575	0.544, 0.608	<0.001
16		0.575	0.544, 0.608	<0.001
17		0.575	0.544, 0.608	<0.001
18		0.574	0.543, 0.607	<0.001
19		0.574	0.542, 0.607	<0.001
20		0.575	0.544, 0.609	<0.001
21		0.575	0.544, 0.608	<0.001
22		0.575	0.543, 0.608	<0.001
23		0.573	0.542, 0.606	<0.001
24		0.576	0.544, 0.609	<0.001
25		0.574	0.543, 0.607	<0.001
1	Heart Rhythm (Non Sinus)	1.271	1.169, 1.382	<0.001
2		1.272	1.170, 1.383	<0.001
3		1.273	1.171, 1.384	<0.001
4		1.273	1.171, 1.384	<0.001
5		1.263	1.161, 1.373	<0.001
6		1.274	1.171, 1.385	<0.001
7		1.267	1.165, 1.378	<0.001
8		1.261	1.160, 1.372	<0.001
9		1.267	1.165, 1.378	<0.001

10		1.265	1.163, 1.375	<0.001
11		1.263	1.161, 1.373	<0.001
12		1.264	1.162, 1.375	<0.001
13		1.261	1.160, 1.372	<0.001
14		1.265	1.164, 1.376	<0.001
15		1.267	1.165, 1.378	<0.001
16		1.266	1.165, 1.377	<0.001
17		1.268	1.166, 1.379	<0.001
18		1.266	1.164, 1.377	<0.001
19		1.267	1.165, 1.377	<0.001
20		1.265	1.164, 1.376	<0.001
21		1.262	1.161, 1.373	<0.001
22		1.262	1.160, 1.372	<0.001
23		1.256	1.155, 1.366	<0.001
24		1.268	1.166, 1.379	<0.001
25		1.266	1.164, 1.376	<0.001
1	Chronological Age (<=50)	1.054	1.046, 1.062	<0.001
2		1.054	1.046, 1.062	<0.001
3		1.054	1.046, 1.062	<0.001
4		1.054	1.046, 1.062	<0.001
5		1.054	1.046, 1.062	<0.001
6		1.054	1.046, 1.062	<0.001
7		1.054	1.046, 1.062	<0.001
8		1.054	1.046, 1.062	<0.001
9		1.054	1.046, 1.062	<0.001
10		1.054	1.046, 1.062	<0.001
11		1.054	1.046, 1.062	<0.001
12		1.054	1.046, 1.062	<0.001
13		1.054	1.046, 1.062	<0.001
14		1.054	1.046, 1.062	<0.001
15		1.054	1.046, 1.062	<0.001
16		1.054	1.046, 1.062	<0.001
17		1.054	1.046, 1.062	<0.001
18		1.054	1.046, 1.062	<0.001
19		1.054	1.046, 1.062	<0.001
20		1.054	1.046, 1.062	<0.001

21		1.054	1.046, 1.062	<0.001
22		1.054	1.046, 1.062	<0.001
23		1.054	1.046, 1.062	<0.001
24		1.054	1.046, 1.062	<0.001
25		1.054	1.046, 1.062	<0.001
1	Chronological Age (>50)	1.126	1.122, 1.130	<0.001
2		1.126	1.122, 1.130	<0.001
3		1.126	1.122, 1.130	<0.001
4		1.126	1.122, 1.130	<0.001
5		1.126	1.122, 1.130	<0.001
6		1.126	1.122, 1.130	<0.001
7		1.126	1.122, 1.130	<0.001
8		1.126	1.122, 1.130	<0.001
9		1.126	1.122, 1.130	<0.001
10		1.126	1.122, 1.130	<0.001
11		1.126	1.122, 1.130	<0.001
12		1.126	1.122, 1.130	<0.001
13		1.126	1.122, 1.130	<0.001
14		1.126	1.122, 1.130	<0.001
15		1.126	1.122, 1.130	<0.001
16		1.126	1.122, 1.130	<0.001
17		1.126	1.122, 1.130	<0.001
18		1.126	1.122, 1.130	<0.001
19		1.126	1.122, 1.130	<0.001
20		1.126	1.122, 1.130	<0.001
21		1.126	1.122, 1.130	<0.001
22		1.126	1.122, 1.130	<0.001
23		1.126	1.122, 1.130	<0.001
24		1.126	1.121, 1.130	<0.001
25		1.126	1.122, 1.130	<0.001

Supplement 40: EHRA AI Checklist

(10)

THE EHRA AI checklist for reporting, reading and understanding AI studies in clinical EP				
**Item #**	**Category/Section**	**Explanation**	**Rationale**	**Page #**
	**TITLE**			
i)	***Title***	Include clear terms to identify the study as using artificial intelligence, machine learning or other specific terms	To facilitate paper retrieval the terms artificial intelligence/machine learning/neural network in the context of EP should be used	1
	**INTRODUCTION**			
1	**Intended clinical use**	Clearly describe the intended use and where in clinical workflow the model can be used and the objective of the study	To provide clear information of the clinical context in which to use the suggested AI solution in the context of EP	5-6
2	**Clinical benefit**	Added benefit of AI compared to standard clinical care (gold standard)	To explain how the AI is performing compared to clinical care (gold standard/standard practice) to better evaluate the performance of the AI model and its potential added benefit	5-6
	**METHODS**			
3	**Data Collection**	Describe how data was collected	To provide a clear description of the dataset generation process, for example was data retrospectively of prospectively collected, from a single center, or multicenter?	6-7,sup 4
4	**Source (of data)**	Describe the study design or source of input data and how it was acquired	To describe how the input data was acquired including the study design - for example RCT, cohort, registry data	6
5	**Development data set (model training data set)**	Describe the data set	To describe the data set that was used for training of the model (i.e 12-lead ECGs from a specific population)	7-8
6	**Participants**	Describe the participants in the data sets, including eligibility criteria (inclusion and exclusion criteria).	Flow chart of participants (or table) suggested	7-8
7	**Comparator**	Provide clear definition of how the gold standard was collected.<br>Clearly describe the gold standard and ground truth including limitations.	To describe in detail how ground truth the model was trained on was established - human interaction, consensus, review type). For example, how was the diagnosis of atrial fibrillation established (12 lead ECG interpreted by independent electrophysiologists)	7
8	**Validation data set**	Describe the validation data set, in particular defining the data set split.	To describe in detail the data set that was used for validating the model, and the rationale bases on which the whole dataset was split and how.	7-8
9	**Sample Size**	Explain how the study size was arrived at.	For supervised models: Focus in particular on the training set including number of positives/negatives and the use of data augmentation/reduction (legitimization). For unsupervised models: focus on the number of participants	7-8
10	**Outcome**	Clearly define standardized and reproducible outcome of clinical relevance.	To clearly describe the outcome, for example the accuracy of a specific algorithm	7

11	**Data type (source)**	Clearly describe the data type for the study, including pre-processing	To describe the data used (i.e., ECG, image, EGM, omics, EHR..) and its specification used to train and validate the model (i.e., was the information from an ECG in a image or a digital format)	6-8,sub 4
12	**Data Preparation**	*Input data handling, data augmentation and selection prior to analysis by the AI system, application of techniques to prevent data leakage.*	To describe every step of handling the data (i.e., was the data reused at any time in the model, like using one ECG to provide several data points)	8,sub 5,6
13	**Balanced groups**	Clearly state how/if groups were balanced	To describe in detail the data set that was used for validating the model, and the rationale bases on which the whole dataset was split and how.	7
14	**Data issues (missingness / poor data / duplication / outliers)**	Describe how handling of data of poor quality/noise/missing data was performed	To provide information about possible issues in the utilized data, as well as how these were identified and handled. It should also be specified if there was a minimum standard for quality required for the input data, and where this standard was not achieved, how this was handled	sub 5,6
15	**Feature engineering (extraction/ selection/reduction)**	If features are used, feature selection should be described including by whom features were extracted.	To describe the process of feature selection (i.e., handcrafted or automatically generated), as well as the strategy adopted to reduce their number (i.e., threshold on cumulative explained variance)	6,sub 4
	**REGULATORY**			
16	**Legal framework**	Clearly state if the software has been approved by legal authorities, e.g. Certificate of conformity (EU) or FDA approval or other, and add further details, where appropriate (e.g. risk class).	To provide information about the certification process undergone by the AI software specific version, and associated risk class for its use as declared by the manufacturer	7
17	**Explainability**	Is the AI model explainable on the patient level or on a population level.	To provide a description of the methodology used to provide model explainability	8-9
18	**Ethical approval**	Provide information on ethical approval of the study.	To clearly describe which entity evaluated and released the ethical approval for the study	7
19	**Fairness**	Describe inclusion of relevant groups in the dataset	To describe the efforts made to ensure fairness in the study, including for example age, ethnicity and gender	6-7
	**OPEN SCIENCE**			
20	**Data availability/ Code sharing**	Is the data available on a public website? Is the code available?	To provide details on how to access the anonymized data used for training/validating the model, as well as code sharing	4
21	**Trial registration**	In case of a trial, clearly state if and where the trial is registered.	Provide the number and the reference for the trial registration.	/
	**RESULTS**			
22	**Participants**	Baseline demographics (internal and external validation data).	*To clearly describe the participant demographics in the study/trial/inclusion to perform internal validation of the AI model, as well as the dataset used for external validation."*	11

23	**Training performance**	Provide results from the training data set	To provide results using proper metrics describing the model performance when applied to the training set, in order to provide a reference for the expected model performance and allow overfitting assessment in non-externally validated studies	11
24	**Internal validation**	The results from the testing data set	To provide results using proper metrics describing the model performance when applied to the validation set, as obtained from the same population/hospital/study/equipment	11
25	**External validation**	The results from the external validation data set	To provide results using proper metrics describing the model performance when applied to a validation set obtained from a different population/hospital/study/equipment	11
26	**Model performance Internal and external validation**	Choose appropriate metric selection for reporting	"To provide appropriate metrics (threshold dependent or independent), for example: AUC/Sensitivity/Specificity/NPV/PPV/F1/Uncertai nty Failing cases"	11
27	**Performance errors**	Analysis of performance errors and how they were identified	To provide description about how errors in the model were detected, possible explanations, and potential corrections taken	12
28	**Performance compared to classic statistical methods**	What did the model add?	To provide a comparison with a regular statistical model if applicable, potentially using net reclassification indices (i.e., what would have been the results of a regression model compared to the AI-algorithm)"	/
29	**Generalizability**	Discuss the level of generalizability of the obtained results.	To discuss how and within which limits the obtained results could be generalized to a more general population, with regards to internal and external validation data sets	15
	**CONCLUSION**			
ii)	**Conclusion**	Is the conclusion supported by the dataset?		17-18

References

1. WHO. ATC/DDD. [Online].; 2025 [cited 2025 June 17. Available from: https://atcddd.fhi.no/.
2. Rozanski A, Han D, Blaha M, Gransar H, Friedman J, Hayes S, et al. Association between hypercholesterolemia and mortality risk among patients referred for cardiac imaging test: Evidence of a "cholesterol paradox?" Prog Cardiovasc Dis. 2022 October; 74: 60-69.
3. Nowak M, Niemczyk M, Florczyk M, Kurzyna M, Pączek L. Effect of Statins on All-Cause Mortality in Adults: A Systematic Review and Meta-Analysis of Propensity Score-Matched Studies. J Clin Med. 2022 September; 11(19): 5643.
4. Little R. Missing-data adjustments in large surveys. J Business & Econ Stat. 1988 July; 6: :287-296.
5. Li K, Meng X, Raghunathan T, Rubin D. Significance levels from repeated p-values with multiply-imputed data. Statistica Sinica. 1991;: 65-92.
6. Grund S, Ludtke O, Robitzsch A. Pooling ANOVA Results From Multiply Imputed Datasets. Methodology. 2016 July; 12(3).
7. White I, Royston P. Imputing missing covariate values for the Cox model. Statistics in Medicine. 2009 July; 28(15): 1982-1998.
8. Rubin D. Multiple imputation. In van Buuren S. Flexible Imputation of Missing Data, Second Edition. New York: Chapman and Hall/CRC; 2018. p. 29–62.
9. Attia Z, Friedman P, Noseworthy P, Lopez-Jimenez F, Ladewig D, Satam G, et al. Age and Sex Estimation Using Artificial Intelligence From Standard 12-Lead ECGs. Ciculation Arrhythmia and Electrophysiology. 2019 September; 12(9): e007284.
10. Svennberg E, Han J, Caiani E, Engelhardt S, Ernst S, Friedman P, et al. State of the Art of Artificial Intelligence in Clinical Electrophysiology in 2025: A Scientific Statement of the European Heart Rhythm Association (EHRA) of the ESC, the Heart Rhythm Society (HRS), and the ESC Working Group on E-Cardiology. EP Europace. 2025 May; 27(5).
